# BRD8 Guards the Pluripotent State by Sensing and Maintaining Histone Acetylation

**DOI:** 10.1002/advs.202409160

**Published:** 2024-12-10

**Authors:** Li Sun, Xiuling Fu, Zhen Xiao, Gang Ma, Yibin Zhou, Haoqing Hu, Liyang Shi, Dongwei Li, Ralf Jauch, Andrew Paul Hutchins

**Affiliations:** ^1^ Department of Systems Biology Southern University of Science and Technology Shenzhen 518055 China; ^2^ School of Biomedical Sciences Li Ka Shing Faculty of Medicine The University of Hong Kong Hong Kong SAR China; ^3^ Centre for Translational Stem Cell Biology Hong Kong SAR China; ^4^ Key Laboratory of Biological Targeting Diagnosis Therapy and Rehabilitation of Guangdong Higher Education Institutes The Fifth Affiliated Hospital of Guangzhou Medical University Guangzhou 510799 China

**Keywords:** chromatin, epigenetics, histone acetylation, pluripotent stem cells

## Abstract

Epigenetic control of cell fates is a critical determinant to maintain cell type stability and permit differentiation during embryonic development. However, the epigenetic control mechanisms are not well understood. Here, it is shown that the histone acetyltransferase reader protein BRD8 impairs the conversion of primed mouse EpiSCs (epiblast stem cells) to naive mouse ESCs (embryonic stem cells). BRD8 works by maintaining histone acetylation on promoters and transcribed gene bodies. BRD8 is responsible for maintaining open chromatin at somatic genes, and histone acetylation at naive‐specific genes. When *Brd8* expression is reduced, chromatin accessibility is unchanged at primed‐specific genes, but histone acetylation is reduced. Conversely, naive‐specific genes has reduced repressive chromatin marks and acquired accessible chromatin more rapidly during the cell type conversion. It is shown that this process requires active histone deacetylation to promote the conversion of primed to naive. This data supports a model for BRD8 reading histone acetylation to accurately localize the genome‐wide binding of the histone acetyltransferase KAT5. Overall, this study shows how the reading of the histone acetylation state by BRD8 maintains cell type stability and both enables and impairs stem cell differentiation.

## Graphical abstract

Epigenetic control is a critical process required to control cell type. However, the mechanisms remain incompletely understood. This study reveals how BRD8 regulates epigenetic control in embryonic stem cells, blocking the conversion of primed EpiSCs to naive ESCs by maintaining histone acetylation. BRD8 localized the histone acetyltransferase KAT5 to maintain cell type and its loss improved stem cell conversion.

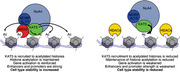

## 1 Introduction

Despite containing the same DNA sequence cells can execute specialized functions and form unique cell types. Changes in cell type are prevalent during embryonic development when the fertilized zygote goes through an elaborate developmental program as cells become progressively restricted to specific cell fates.^[^ ^1^ ^]^ Mechanistic control of cell fates remains unclear but is thought to be caused by changes in epigenetic control of DNA and chromatin driven by the activity of cell type‐specific transcription factors (TFs).^[^ ^2^, ^3^ ^]^ However, while some TFs are expressed in a cell type‐specific pattern, they cooperate with other cell type‐independent TFs and epigenetic factors that are often constitutively expressed.^[^ ^4^ ^]^ Consequently, epigenetic factors that erect barriers to cell‐type conversions are hard to identify and barriers that resist cell‐type conversion remain only sporadically described.

Much work has been devoted to the reprogramming of somatic cells to induced pluripotent stem cells, which has revealed a wide array of epigenetic barriers.^[^ ^5^ ^]^ However, somatic cell reprogramming is a major cell type conversion with many changes that represents a reversion spanning the majority of development. A more amenable model is the interconversion of naïve embryonic stem cells (ESCs) and primed epiblast stem cells (EpiSCs) in mouse. The naïve ESCs resemble the early pre‐implantation blastocyst, while the primed EpiSCs are more reminiscent of late epiblast post‐implantation and early gastrulation.^[^ ^6^, ^7^ ^]^ Naïve and primed cells have distinct culture media signalling requirements, with naïve cells requiring BMPs and LIF, and primed cells needing Activin A and FGF‐signalling.^[^ ^8^ ^]^ Attempting to culture naïve cells in primed conditions or vice versa results in a mixture of differentiation and/or cell death.

Interconversion of naïve and primed cell types can nonetheless be performed by modulating cell culture conditions, and in the absence of transgenes. The process is relatively efficient going from naïve‐to‐primed, but usually inefficient going from primed‐to‐naïve.^[^ ^8^ ^]^ Naïve and primed states share overlapping gene regulatory modules, for example, OCT4 and SOX2 are active in both cell types.^[^ ^9^, ^10^, ^11^, ^12^ ^]^ Yet they also have divergent regulatory programs, for example, the TFs OTX2, OCT6, and JUN are active in EpiSCs,^[^ ^12^, ^13^ ^]^ while ESRRB, TFCP2L1, and KLF2, 4, and 5 are active and specific to ESCs.^[^ ^14^, ^15^, ^16^, ^17^ ^]^ Potentially there are epigenetic roadblocks that stop the EpiSCs from reverting to an earlier developmental stage.^[^ ^18^, ^19^ ^]^ There is also evidence of divergent routes when EpiSCs convert to ESCs,^[^ ^11^, ^20^, ^21^ ^]^ and multiple intermediate stages between naïve and primed cell types.^[^ ^7^ ^]^ Epigenetic barriers erect roadblocks that impair conversion.^[^ ^18^, ^22^, ^23^, ^24^ ^]^ However, the full nature of these roadblocks remains unclear, and the mechanisms that fix cell fate and resist conversions remain incompletely understood.^[^ ^2^ ^]^

In this study, we set out to understand the epigenetic barriers that prevent the cell fate conversion from primed EpiSCs to naïve ESCs. We focused on the bromodomain family of epigenetic co‐factors as several family members have been implicated in cell type conversions,^[^ ^25^, ^26^, ^27^, ^28^, ^29^, ^30^, ^31^ ^]^ and we speculated that bromodomain‐family proteins have a general role in modulating cell fate. We discovered a role for the bromodomain‐containing protein BRD8 in blocking cell‐type conversions. BRD8 binds to acetylated histones,^[^ ^32^, ^33^, ^34^ ^]^ and is a member of the NuA4 protein complex that has histone acetyltransferase activity that promotes gene expression.^[^ ^35^ ^]^ Reduced expression of *Brd8* promoted the transition of EpiSCs to ESCs in the primed‐to‐naïve conversion. BRD8 binds to naïve‐specific gene loci, modulates epigenetic modifications at those loci, and then influences cell type transition. This effect was associated with changes in the epigenetic state of the cells caused by the loss of the activator chromatin marks H3K27ac and H3K4me1, and the reduction of the repressive chromatin marks H3K27me3 and H3K9me3 in the transcribed regions of primed‐specific genes. Concomitantly deposition of the variant histone H2AZ was disrupted. Mechanistically cell type conversion required the action of histone deacetylases, and a catalytically active histone acetyltransferase KAT5 (TIP60).

## 2 Results

### 2.1 *Brd8* Impairs the Conversion of EpiSCs to ESCs

To explore the epigenetic factors that control the cell fate conversion of EpiSCs to ESCs, we designed a small‐scale screen to capture genes that influence the conversion. First, we generated OG2‐GFP EpiSCs from OG2 mouse embryos. These cells have multiple copies of the *Pou5f1* locus with GFP (green fluorescent protein) with the EpiSC‐specific proximal enhancer deleted^[^ ^8^, ^36^, ^37^ ^]^ (Figure S1a, Supporting Information). Due to the deletion of the proximal enhancer, they express GFP only when the naïve pluripotency gene expression network is active, in ESCs.^[^ ^20^, ^38^ ^]^ We next modified the culture conditions of the primed‐to‐naive conversion to reduce the efficiency, so that they are still capable of converting to ESCs but do so inefficiently to make it easier to identify factors that improve the conversion.

**Figure 1:**
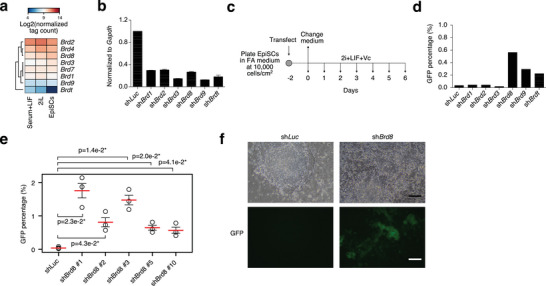
Bromodomain proteins block the conversion of EpiSCs to ESC. a) Heatmap of the expression levels of Bromodomain‐family proteins in ESCs grown in naïve Serum+LIF or groundstate naive 2iLIF (2iL), and in primed EpiSCs. b) RT‐qPCR for the indicated bromodomain‐proteins, in the indicated shRNA knockdowns in EpiSCs. RNA is normalized to *Gapdh*. This experiment was repeated three times. Error bars are standard error of the means. c) Schematic of the reprogramming of EpiSCs to ESCs in the primed‐to‐naïve transition. The EpiSCs used for the conversion contain the OG2‐GFP reporter which is only activated in ESC state. d) Percent of GFP+ cells as counted by FACS (Fluorescence activated cell sorting) on day 6 of the conversion of EpiSCs to ESCs in the indicated knockdowns. The experiment was performed twice, and the bar represents the mean of the two replicates. e) Dot plot showing the GFP percentages of cells at day 5 of a primed‐to‐naïve transition in cells transfected with a control sh*Luc*, or an shRNA targeting *Brd8*. The experiment was performed three times in biological replicate. Significance is from a two‐sided Welch's t‐test. * indicates a p‐value < 0.05. f) Bright‐field and GFP images of colonies in the indicated knockdowns at day 5 of the primed‐to‐naïve conversion. Scale bar = 20 µm.

Bromodomain proteins have been implicated in cell type conversions, particularly BRD4,^[^ ^27^ ^]^ but other BRD proteins also have roles in cell type control.^[^ ^26^, ^28^, ^29^, ^30^, ^31^ ^]^ We reasoned that bromodomain proteins in general might have systematic roles in (embryonic) cell type conversions. Hence, we individually knocked down bromodomain‐family genes that were expressed in ESCs and EpiSCs (**Figure** **1**a,b). We then converted the cells to the naïve state (Figure 1c) and measured the percentage of GFP+ ESCs by FACS (Figure 1d; Figure S1a, Supporting Information). Note that the knockdown of *Brd4* and *Brd7* was lethal in the primed‐to‐naïve conversion system and no cells remained after 4 days. This agrees with a previous study which showed that the knockdown of *Brd4* is incompatible with the naïve pluripotent state.^[^ ^39^ ^]^ Several bromodomain family factors influenced the reversion of EpiSCs to ESCs, including *Brd8*, *Brd9*, and *Brdt* (Figure 1d). The last was surprising as *Brdt* is expressed at low levels in both EpiSCs and ESCs (Figure 1a). BRD9 has previously been implicated in controlling human pluripotency through a non‐canonical BAF protein complex,^[^ ^26^ ^]^ and its inhibition promoted the conversion of ESCs to an EpiSC‐like state.^[^ ^25^ ^]^ Our data suggests that reduced *Brd9* promotes the conversion of EpiSCs to ESCs. However, a function for *Brd8* in embryogenesis has not been reported in any embryonic model system. Hence we focused on *Brd8*, as it also caused the clearest improvement in the generation of GFP+ ESC‐like cells (Figure 1d). We confirmed this effect for five different *Brd8* shRNAs, and all five led to an increase in the percentage of GFP+ cells (Figure 1e,f; Figure S1b, Supporting Information). This improvement in cell type conversion agrees with genome‐wide knockout/knockdown studies for the conversion of EpiSCs to ESCs, which placed *Brd8* as either rank 6,^[^ ^19^ ^]^ or rank 678^[^ ^9^ ^]^ as the best sgRNAs.

### 2.2 Loss of *Brd8* Promotes the Suppression of Somatic Genes and Expression of Naïve‐Specific Genes

We next examined the conversion of EpiSCs to ESCs when *Brd8* was reduced. As our cells are custom‐derived EpiSC lines, we took advantage of the RNA‐seq data to confirm that the EpiSCs we derived from OG2 mice were correct and correlated closely with previously described EpiSCs. RNA‐seq of the EpiSCs generated here closely correlated with EpiSC RNA‐seq data from multiple studies from independent labs (Figure S2a, Supporting Information).^[^ ^40^, ^41^, ^42^, ^43^, ^44^, ^45^, ^46^ ^]^ They also had typical EpiSC‐like cell morphology (Figure S2b, Supporting Information). RNA‐seq ESC‐OG2 cells derived from EpiSCs also closely correlated with ESCs (Figure S2a, Supporting Information), and expressed GFP from the OG2 reporter, as expected (Figure S2c, Supporting Information). Additionally, primed and naïve‐specific marker genes were correctly expressed in our lines versus other reported EpiSCs and ESCs (Figure S2d, Supporting Information). RT‐qPCR confirmed that the EpiSCs specifically expressed the EpiSCs specific markers *Otx2*, *T*, and *Vim*, had lower levels of the ESC/EpiSC markers *Pou5f1*, *Sall4* and *Nanog*, and did not express the naive‐specific gene *Tfcp21l1* (Figure S2e, Supporting Information).

**Figure 2:**
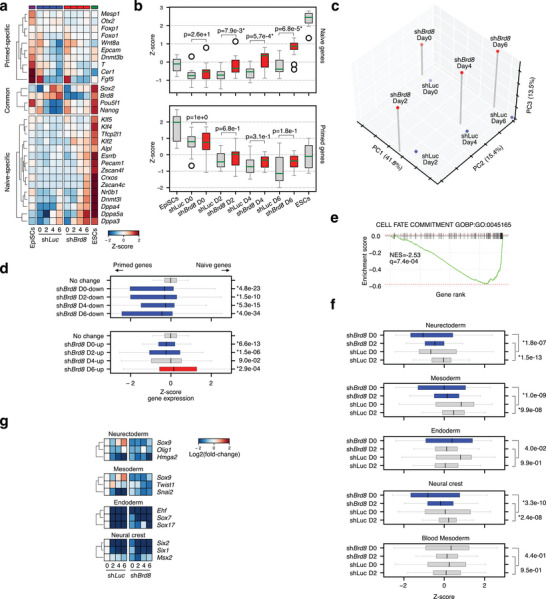
Knockdown of *Brd8* causes the accelerated downregulation of primed and somatic genes and the late‐stage activation of naïve‐specific genes. a) Heatmap showing RNA‐seq data for a selection of marker genes in EpiSCs, 2iL grown ESCs, and in a primed‐to‐naïve conversion time course in cells transfected with sh*Luc* as a control, or sh*Brd8*. b) Boxplots of the marker genes’ expression levels, as in panel a. Significance is from a two‐sided Welch's t‐test. * indicates p<0.01. D = day. c) Principal component analysis (PCA) of the RNA‐seq time course for the primed‐to‐naïve conversion in sh*Luc* or sh*Brd8*‐transfected cells. d) Boxplots of the sum of Z‐scores for primed or naïve‐specific genes (as defined in Figure S3a,b, Supporting Information) versus down‐regulated (top boxplot) or up‐regulated (bottom boxplot) genes in the sh*Brd8* knockdown. Significance is from a two‐sided Welch's t‐test versus the genes that had no change. * indicates a p‐value < 0.05. e) GSEA plot for the down‐regulated genes at day 0 of the primed‐to‐naïve conversion in sh*Luc* versus sh*Brd8* cells. A term was considered significant if it had an absolute normalized enrichment score (NES) of at least 1.5 and a q‐value of <0.01. f) Boxplots showing sums of Z‐scores for the indicated germ lineage‐specific genes as defined in.^[^ ^4^ ^]^ Significance is from a two‐sided Welch's t‐test versus the genes that had no change. * indicates a p‐value < 0.05. g) Heatmap of the fold‐change for selected germ lineage‐specific genes. Fold‐change is calculated relative to day 0 sh*Luc* transfected cells.

Time course RNA‐seq in cells transfected with sh*Luc* (Luciferase control) or sh*Brd8* confirmed the up‐regulation of naive‐specific genes when *Brd8* was knocked down, particularly at days 2–6 (**Figure** **2**a,b). During the primed‐to‐naïve conversion, specific marker genes for the naïve state were up‐regulated as early as day 4, for example, *Tfcp2l1*, *Dppa5a*, *Dppa2*, and *Esrrb* were all upregulated in the *Brd8* knockdown cells (Figure S2f,g, Supporting Information). Some primed‐specific genes were down‐regulated slightly earlier, for example, *T*, *Dchs1*, and *Otx2*. However, other primed‐specific genes, *Ets1* or *Fgf8*, were similar between the *Luc* and *Brd8* two knockdowns. (Figure 2a,b; Figure S2f,g, Supporting Information). Interestingly, principal component analysis (PCA) of the RNA‐seq data indicated that while the overall trajectory was accelerated in the sh*Brd8* transfected cells, the change was already apparent upon shRNA transfection at day 0, which is 2 days after the addition of the shRNA, but before a change in cell culture medium (Figure 1b and Figure 2c). We wondered if this day 0 change was representative of a general suppression of primed‐specific genes and was a systematic phenomenon. Hence, we defined sets of primed‐ and naïve‐specific genes by looking at genes that were 2‐fold up or downregulated between ESCs and EpiSCs (Figure S3a,b and Table S1, Supporting Information). The expression of these two gene sets supported an accelerated up‐regulation of naïve‐specific genes, and accelerated suppression of primed‐specific genes (Figure 2d). Indeed, primed‐specific genes were consistently downregulated at all time points, while naive‐specific genes were only upregulated at day 6 (Figure 2d). This was supported by GSEA, as the down‐regulated genes in the sh*Brd8*‐transfected day 0 cells were associated with GO terms related to differentiation such as “cell fate commitment”, and neuron, heart, and spinal cord differentiation (Figure 2e; Figure S3c, Supporting Information). Interestingly, GSEA at day 0 for the up‐regulated genes suggested blastocyst formation, LIF response, and regulation of stem cell populations as significantly up‐regulated (Figure S3c, Supporting Information). This suggests that even as early as day 0, the EpiSCs have suppressed some somatic genes, and activated embryonic genes.

EpiSCs are poised to differentiate and express low levels of differentiation‐related and somatic genes, particularly genes expressed early in gastrulation. To confirm that somatic genes are reduced in the *Brd8* knockdowns we utilized our datasets of germ layer‐specific genes,^[^ ^4^ ^]^ and scored the overall level of expression of these gene sets in the *Luc* controls and *Brd8* knockdowns (Figure 2f; Figure S3d, Supporting Information). Genes for most germ lineage‐specific genes were significantly downregulated in the sh*Brd8* knockdowns at early time points (Figure 2f; Figure S3d, Supporting Information). These results are exemplified by the downregulation of germ layer‐specific genes as early as day 0 in the *Brd8* knockdown cells (Figure 2g). Overall, this data suggests that reduced *Brd8* destabilizes the expression of differentiation‐specific genes in the earliest stages of conversion to ESCs which is succeeded by an acceleration in the activation of naïve‐specific genes in the late stage. We conclude that BRD8 acts as an epigenetic rheostat to maintain cell fates while its depletion derails the status quo.

### 2.3 Reduced *Brd8* Promotes Chromatin Changes

**Figure 3:**
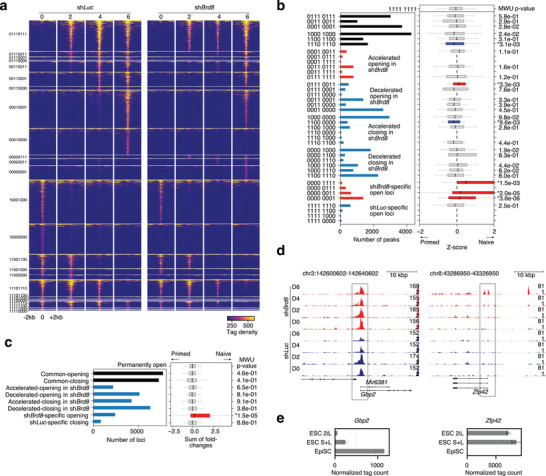
Reduced *Brd8* expression leads to changes in chromatin accessibility. a) Pileup heatmaps for selected ATAC‐seq accessibility data showing the clusters of chromatin loci that change in the indicated conditions according to the binary key on the left‐hand side. For the binary key, 1 indicates the presence of an open “peak” of binding, while 0 indicates no peak detected. The full heatmap showing all clusters is in Figure S4a (Supporting Information). b) Combined plot showing the number of peaks in each category of binding (As in panel a) (left side), and the expression of genes with a TSS within 2000 bp of the chromatin accessibility locus (right side). The genes were defined as a Z‐score based on their expression in EpiSCs versus ESCs (See Figure S3a,b, Supporting Information). A positive Z‐score indicates a gene more likely to be high in naïve ESCs, and a negative Z‐score more likely to be high in EpiSCs. Significance is from a Mann‐Whitney U test versus the 1111 1111 category of binding. * indicates a p‐value < 0.05. c) Bar chart showing the number of peaks in the categories defined in panel b, and the sum of fold‐changes for all genes for naïve versus primed cells. Significance is from a Mann‐Whitney U test versus the permanently open category. * indicates a p‐value < 0.05. d) Genome pileup plots at the primed gene *Gbp2*, and the naïve gene *Zfp42*. e) Bar chart showing gene expression levels of the primed‐specific gene *Gbp2* and the naïve‐specific gene *Zfp42*, from RNA‐seq data for primed and naïve cells grown in 2iLIF (2iL), serum+LIF (S+L) or EpiSC culture conditions.

To explore the changes in the chromatin state, which can often precede changes in gene expression,^[^ ^47^ ^]^ we assayed chromatin accessibility using ATAC‐seq,^[^ ^48^ ^]^ and defined open and closed groups as previously described.^[^ ^47^, ^49^ ^]^ The resulting pattern of chromatin changes was complex (**Figure** **3**a; Figure S4a, Supporting Information). We focused on those peaks that were dynamically changing over the time course, which amounted to 36203 loci. We divided those peaks into several categories based on their pattern in the sh*Brd8* knockdown (Figure 3b,c). Knocking down *Brd8* had varied effects on chromatin accessibility and the changes were equally distributed between accelerated and decelerated opening or closing (Figure 3c). This was curious and suggests that reduced *Brd8* is increasing overall cellular plasticity, rather than influencing the chromatin for a specific cell type. To explore this further, we associated the nearby genes with their expression level in naïve or primed cells using a Z‐score (Figure 3b,c). This will reveal if a category of chromatin changes is associated with naïve or primed‐specific genes. Despite the major changes in chromatin, only one category of chromatin changes was significantly associated with naïve‐specific genes, sh*Brd8*‐specific open loci (Figure 3b,c). This is reflected in the accessibility of chromatin at naïve‐specific genes, such as *Zfp42*, *Dppa5a, App4, and Nr0b1* loci that were all open at day 6 when *Brd8* was knocked down (Figure 3d,e; Figure S4b,c, Supporting Information). Curiously, no category of chromatin change was significantly associated with primed‐specific genes (Figure 3c), suggesting that changes in chromatin accessibility is not a driving force in the suppression of somatic genes. Indeed, primed‐specific genes tended to retain open chromatin in the *Brd8* knockdowns, and in some cases even had increased chromatin accessibility in the *Brd8* knockdown cells at day 6. For example, the primed genes *Gbp2*, *Dab1*, *Aplpr*, and *Flt1* all had chromatin accessibility that was similar to the control sh*Luc* cells or increased in the *Brd8* knockdowns (Figure 3d,e; Figure S4d,e, Supporting Information). These results, combined with the RNA‐seq results, suggest that chromatin accessibility changes only underly the increases in naïve‐specific gene expression. Primed‐specific genes, conversely, retain open chromatin when *Brd8* was knocked down. This suggests two distinct epigenetic mechanisms are regulating naïve and primed genes.

### 2.4 BRD8 and the NuA4 Complex Associates with Naïve‐Specific Transcription Factors in ESCs

**Figure 4:**
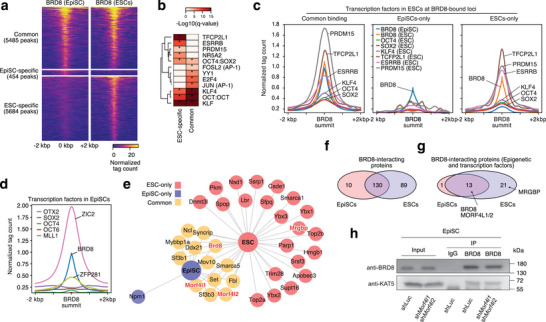
BRD8 is a member of a novel protein complex in EpiSCs and ESCs, and associates with naïve TFs. a) Heatmap pileups for CUT&Tag of BRD8 in EpiSCs and 2iL grown ESCs. Peaks were divided into those loci common to ESCs and EpiSCs (Common‐binding), and ESC‐ and EpiSC‐specific loci. b) TF motif discovery in the EpiSC‐specific group versus the BRD8 common binding peaks. c) Cumulative pileup plots for a selection of ESC ChIP‐seq data. ChIP‐seq data is from GSE11431,^[^ ^53^ ^]^ and GSE73692.^[^ ^52^ ^]^ The data is centered on the BRD8 binding peak for the groups of binding as defined in panel a. See also Figure S5a (Supporting Information) for the full heatmap of all binding factors. d) Cumulative pileup plots for a selection of ESC ChIP‐seq data. Data is from GSE74636,^[^ ^10^ ^]^ GSE73992,^[^ ^54^ ^]^ and GSE93042.^[^ ^55^ ^]^ See also Figure S5b (Supporting Information) for the full heatmap of all binding factors. e) Network of interacting transcription or epigenetic factors detected in the Co‐IP/MS for BRD8 binding partners in EpiSCs and ESCs. Commonly detected peptides are marked in orange, red is specific to ESCs, and blue is specific to EpiSCs. The NuA4 complex members Morf4l1/2 and Mrgbp are indicated. The full table of interacting proteins is in Figure S6a and Table S2 (Supporting Information). f) Venn diagram of the overlap of all BRD8 interacting proteins in ESCs and EpiSCs. g) Venn diagram, as in panel f, but only including transcription and epigenetic factors. h) Western blot of Co‐IP with the indicated antibodies (left side) and immunoprecipitations (IPs) in EpiSCs. The EpiSCs were transfected with shRNAs targeting *Luc* (control) or both *Morf4l1* and *Morf4l2*. The input is shown for comparison.

We next looked at the genome‐wide binding of BRD8 using CUT&Tag.^[^ ^50^ ^]^ We first took this opportunity to confirm the OG2‐containing EpiSC and ESC lines. The OG2 cassette is present in ≈20 copies in the OG2 mice genome,^[^ ^36^ ^]^ and this is reflected in a general enrichment of the background at the *Pou5f1* locus (Figure S5a, Supporting Information). Surprisingly, genome‐wide BRD8 binding in EpiSCs was a subset of the binding pattern seen in ESCs, with 5485 peaks common to EpiSCs and ESCs, 5684 loci specific to ESCs and only 454 loci specific to EpiSCs (**Figure** **4**a). Motif discovery in the common and ESC‐specific peaks indicated that BRD8 was primarily associated with motifs related to pluripotency (OCT4:SOX2, KLF), or were naïve‐specific transcription factors, such as TFCP2L1, ESRRB, and PRDM15^[^ ^15^, ^51^, ^52^ ^]^ (Figure 4b). We reanalyzed ChIP‐seq data in ESCs matching the predicted motifs in Figure 4b, including the key pluripotent TFs OCT4, SOX2, and KLF4, along with the naïve‐specific TFs PRDM15, TFCP2L1 and ESRRB factors in ESCs.^[^ ^52^, ^53^ ^]^ BRD8 was indeed bound at the same loci as TFCP2L1, ESRRB, and PRDM15 in ESCs, (Figure 4c; Figure S5b,c, Supporting Information). BRD8 tended to co‐bind with epigenetic factors (CDK9, MED1, MED12, SMC1A, SMARC4), rather than other transcription factors (Figure S5b,c, Supporting Information). Indeed, for OCT4 and SOX2, two transcription factors that are common to both ESCs and EpiSCs,^[^ ^10^ ^]^ they were not co‐bound at BRD8‐bound loci (Figure S5b,c, Supporting Information). This suggests that BRD8 specifically binds to loci with epigenetic factors and naïve‐specific TFs in ESCs. Indeed, reanalysis of ChIP‐seq data in EpiSCs showed that BRD8 is not associated with OCT4, OCT6, SOX2, or OTX2, and is only bound with the primed pluripotency marker ZIC2 (Figure 4d; Figure S5c,d, Supporting Information).^[^ ^10^, ^54^, ^55^ ^]^

To identify the physical interactions of BRD8, we performed co‐immunoprecipitation and mass spectrometry (Co‐IP/MS) to identify protein‐binding partners for BRD8 in ESCs and EpiSCs. In total we identified 229 significant protein interactions in the two cell types (Figure 4e–g; Figure S6a and Table S2, Supporting Information). The interacting partners were similar in the two cell types with 130 proteins in common and tended to be related to DNA binding (Figure 4f; Figure S6b, Supporting Information). Interestingly, no naïve‐specific TFs were identified in the ESC BRD8‐Co‐IP/MS data, nor was ZIC2 identified in EpiSCs, suggesting that BRD8 is interacting indirectly with these factors (Figure 4d–f; Figure S6a and Table S2, Supporting Information). BRD8 is a member of the histone acetyltransferase NuA4 complex,^[^ ^56^ ^]^ however, only a few NuA4 complex members were identified interacting with BRD8: MORF4L1/2, and MRGBP, the latter of which was only co‐precipitated in ESCs (Figure 4e,g). These data suggest that BRD8 binds with MORF4L1/2 and MRGBP as a peripheral component of the NuA4 complex. However, EpiSCs transfected with shRNAs targeting *Morf4l1&2* did not affect the interaction between BRD8 and the core NuA4 histone acetyltransferase KAT5 as measured by Co‐IP western blot (Figure 4h; Figure S6c, Supporting Information). These data indicate BRD8 is in close cooperation with NuA4 complex and does not require MORF4L1/2 for this interaction.

### 2.5 BRD8 Binds at Promoters and Its Loss Reduces Signals Associated with Active Genes

**Figure 5:**
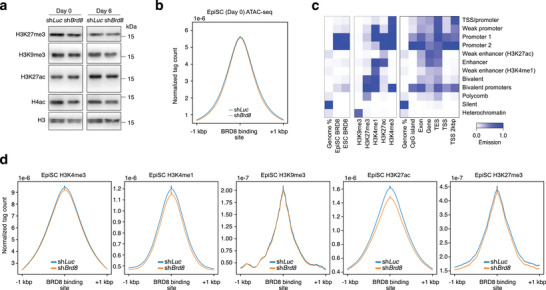
BRD8 regulates chromatin states in EpiSCs. a) Western blot of histone modifications in day 0 and day 6 EpiSCs transfected with the indicated shRNA, and undergoing a primed‐to‐naïve transition. b) Density pileup for ATAC‐seq data centered on BRD8 binding in EpiSCs. ATAC‐seq data is from the day 0 data from cells transfected with shRNAs targeting *Luc* (Control) or *Brd8*. c) ChromHMM heatmap showing the relative abundance of the factor (left heatmap). The 12‐state model (right heatmap) was generated using chromatin ChIP‐seq data in the subsequent section. d) Density pileup for H3K4me3, H3K4me1, H3K9me3, H3K27ac, and H3K27me3 in EpiSCs transfected with the indicated shRNA. The pileups are centered on the BRD8 binding site in EpiSCs.

BRD8 binds acetylated histones through its bromodomain and is a component of the NuA4 chromatin‐modifying complex.^[^ ^35^, ^57^, ^58^ ^]^ Hence, we expected that reduced *Brd8* expression would lead to changes in chromatin. Surprisingly, the western blot of whole cell levels of histone marks did not indicate any changes (**Figure** **5**a), suggesting that modulation of chromatin is context‐specific and not genome‐wide. ATAC‐seq accessibility was unchanged at BRD8‐bound loci (Figure 5b). This was not surprising as we have already shown that loci accessible in primed EpiSCs remain open in the *Brd8* knockdowns (Figure 3d). These results suggest that BRD8 mainly impacts gene expression in a mechanism independent of changes in chromatin accessibility.

To explore this at the histone modification level, we generated genome‐wide data for histone modifications that mark promoters (H3K4me3), active promoters (H3K27ac), promoters and enhancers (H3K4me1), repressed polycomb (H3K27me3) and heterochromatin (H3K9me3) in EpiSCs transfected with a control sh*Luc*, or an shRNA targeting *Brd8*. EpiSCs are a relatively underexplored cell type, and we have generated the first genome‐wide chromatin map. Hence, we used our histone data to generate an EpiSC‐specific ChromHMM 12‐state model,^[^ ^59^ ^]^ to identify the chromatin features BRD8 is bound to. The ChromHMM model displayed chromatin features that have been observed in other cell types, including transcription start sites (TSSs) (H3K4me3), promoters (H3K4me3, H3K27ac, H3K4me1), enhancers (H3K4me1, H3K27ac), bivalent domains (H3K27me3, H3K4me1), polycomb (H3K27me3) and heterochromatin (H3K9me3) (Figure 5c). We also detected two types of atypical enhancers, which we term “Weak Enhancer (H3K27ac)” and “Weak Enhancer (H3K4me1)” as these enhancers appear to be marked only by one of the two typical enhancer marks. There was also an atypical promoter, which we term ‘Promoter 2′ that has reduced emission score for H3K4me1. Using this 12‐state model, BRD8 was primarily associated with promoters, and weakly with bivalent promoters (Figure 5c). BRD8 was not bound to heterochromatin regions (Figure 5c), and it was unsurprising that H3K9me3 was unchanged upon *Brd8* knockdown (Figure 5d). Similarly, when *Brd8* was knocked down the promoter marker H3K4me3 was not substantially reduced (Figure 5d). Instead, the main changes in chromatin marks were in H3K4me1, H3K27ac, and to a lesser extent, H3K27me3 (Figure 5d). Interestingly, the decline in H3K27me3 was in the flanking regions, rather than the actual location of BRD8 binding. BRD8 was previously identified in a NuA4 super complex containing PRC2 proteins in sarcoma,^[^ ^60^ ^]^ however, we did not detect any PRC2 components in the Co‐IP/MS, suggesting changes in H3K27me3 are indirect. As the biggest declines upon *Brd8* depletion were in regions with H3K4me1 and H3K27ac marks (Figure 5d), we reasoned that promoters were being decommissioned upon BRD8 removal.

### 2.6 BRD8 Erects a Barrier by Modulating Chromatin Modifications Inside Gene Bodies

**Figure 6:**
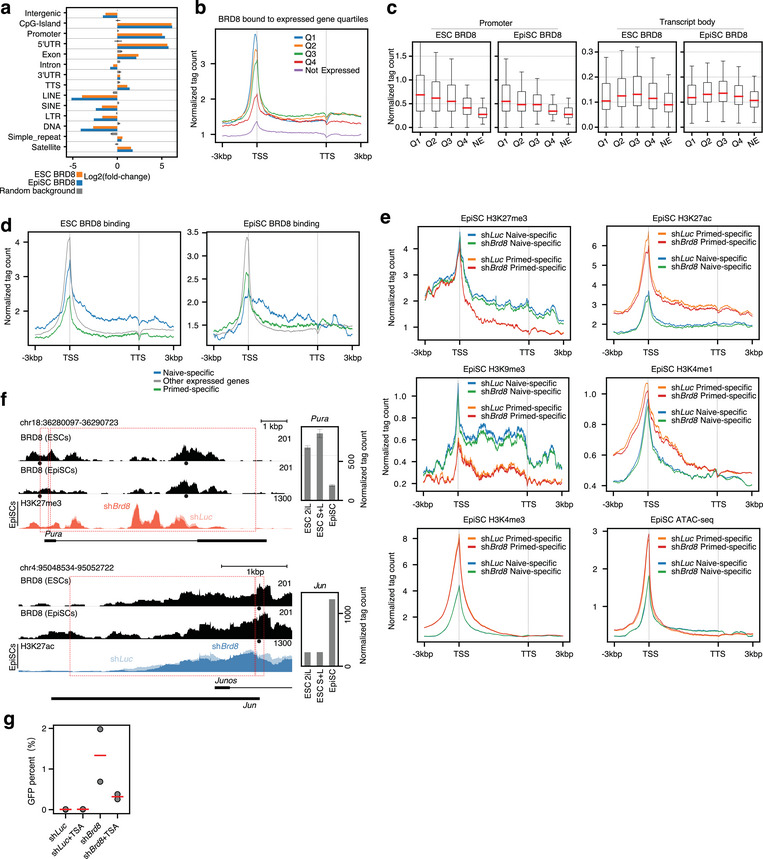
BRD8 regulates chromatin at naïve and primed‐specific genes. a) Bar chart of over or under‐representation of genome features for the binding of BRD8 in ESCs and EpiSCs. Enrichment was calculated with HOMER.^[^ ^89^ ^]^ b) A pileup of BRD8 CUT&Tag signals across all genes in the mouse genome. Genes were divided into expressed (normalized tag count >100) and not expressed. The expressed genes were further subdivided into their expression quartiles, Q1‐Q4. The windows of binding are centered on the TSS and TTS (transcription termination site) and all transcripts are scaled to a uniform length. The flanking 5′ and 3′ regions 3 kbp from the TSS or TTS are shown. For this and all subsequent pileup plots in this figure. c) Box plot showing the ratio of BRD8 signal at the TSS (± 500 bp around the TSS) versus the level in the gene body (500 bp 3′ of the TSS and 500 bp 5′ of the TTS). d) Pileup of BRD8 CUT&Tag signal across naïve, primed‐specific genes versus all other expressed genes (naïve and primed‐specific genes are defined in Figure S3a,b, Supporting Information) in ESCs (left plot) or EpiSCs (right plot). e) Pileups of H3K4me1, H3K4me3, H3K27ac, H3K9me3, H3K27me3, and ATAC‐seq accessibility at naïve and primed‐specific genes (naïve and primed‐specific genes are defined in Figure S3a,b, Supporting Information). f) Genome view showing BRD8 binding at the naïve‐specific gene Klf2 (top plot) and the primed gene Jun (bottom plot) in EpiSCs. Gene expression in primed (EpiSCs) and naïve (2iL and SL) ESCs are shown on the right of the genome view. g) Effect of the HDAC inhibitor TSA on the conversion of primed EpiSCs to naïve ESCs in a primed‐to‐naïve conversion. GFP+ cells were counted by FACS on day 6. The experiment was performed in biological duplicate.

When *Brd8* expression was reduced, H3K27ac declined, along with H3K4me1 and flanking regions of H3K27me3 (Figure 5d). BRD8 tended to concentrate around promoters (Figure 5c and **Figure** **6**a), and binding signals were most prominent close to the TSS (Figure 6a) but were also enriched in the 5′UTRs and exons of transcribed genes (Figure 6a). Pileups of BRD8 binding across all expressed genes in EpiSCs showed that BRD8 was also present in the transcribed gene bodies (Figure 6b). To understand the impact of BRD8 binding on transcription we divided all genes up into expression quartiles, ranked by their total expression level. Based on this, genes were divided into one of five classes: Either not expressed/detectable, or their rank in the expression quartiles (Q1‐Q4). We then measured BRD8 binding density across these transcripts. At the TSS there was a clear correlation between BRD8 binding and higher levels of expression (Figure 6b,c). However, BRD8 binding in the transcript body was more likely to be associated with moderately expressed genes in the Q2 or Q3 quartiles (Figure 6b,c). Even Q4 genes had appreciable levels of BRD8‐binding to their transcript bodies, and only silent genes lacked BRD8 binding (Figure 6b,c).

Cell type‐specifically expressed genes tend to be in the moderate range of overall gene expression levels,^[^ ^4^ ^]^ suggesting BRD8 is regulating cell‐type‐specific genes. Hence, we divided genes in ESCs and EpiSCs into naïve or primed‐specific and all other genes (Figure S3a,b, Supporting Information) and then measured BRD8 binding at these subsets of genes (Figure 6d). The pattern at the TSS and transcribed body was complex: In ESCs, BRD8 binding was high at both the TSS and in the transcript body, while in EpiSCs BRD8 binding at the TSS was high, but both naïve and primed‐specific genes were bound by BRD8 inside the transcript body (Figure 6d). This pattern could be observed at specific genome loci. For example, for the two naïve‐specific genes *Nr0b1*, and *Tfcp2l1*, BRD8 binding at the TSS was reduced in EpiSCs compared to ESCs, but the BRD8 signal in the transcribed bodies was comparable between the two cell types (Figure S7a, Supporting Information). Conversely, at two primed‐specific genes, *Zic2* and *Pdlim7*, BRD8 binding in ESCs was lower than in EpiSCs in both the TSS and in the transcribed gene body (Figure S7b, Supporting Information).

Considering that BRD8 was also spread across transcribed bodies, we looked at the changes in chromatin across the same naïve and primed gene sets when *Brd8* was knocked down. As in the case for the BRD8‐bound loci (Figure 6d), activatory marks H3K27ac, and H3K4me1 levels declined across primed‐specific transcripts when *Brd8* was knocked down (Figure 6e), for example at the primed‐specific gene *Jun* (Figure 6f).^[^ ^13^ ^]^ We looked for potential cross‐talk between H3K27ac and H3K4me1, and there was a significant positive correlation between the two marks (Figure S7c, Supporting Information). This makes sense as BRD8 is associated with promoters marked by both H3K27ac and H3K4me1 (Figure 5c). This potential cross‐talk suggests a weakening of both epigenetic marks at promoters is driven by reduced *Brd8*. For ATAC‐seq, however, accessibility and H3K4me3 were unaltered (Figure 6e). Intriguingly, repressive marks H3K27me3 and H3K9me3 were unaltered at the TSSs, but were reduced at naïve‐specific transcript bodies, for example in the naïve‐specific gene *Pura* (Figure 6f). These data suggest that the ultimate impact of reduced *Brd8* is to disrupt activatory chromatin marks at primed‐specific genes and repressive marks at naïve‐specific genes, thus loosening the overall epigenetic stability by weakening chromatin regulation.

These data suggest that the weakening of H3K27ac at primed‐specific genes underlies the ability of reduced *Brd8* to promote the primed‐to‐naïve transition. Consequently, we reasoned that inhibition of histone deacetylases (HDACs) using the inhibitor TSA would block the action of reduced *Brd8* expression. This was indeed the case, as TSA impaired the percentage of GFP+ cells in the *Brd8* knockdowns (Figure 6g). These data indicate that BRD8 is binding to acetylated histones, and when Brd8 expression is reduced active histone deacetylation is required for the primed‐to‐naïve conversion.

### 2.7 BRD8 Co‐Operates with the Acetyltransferase KAT5 on Primed‐Specific Genes

**Figure 7:**
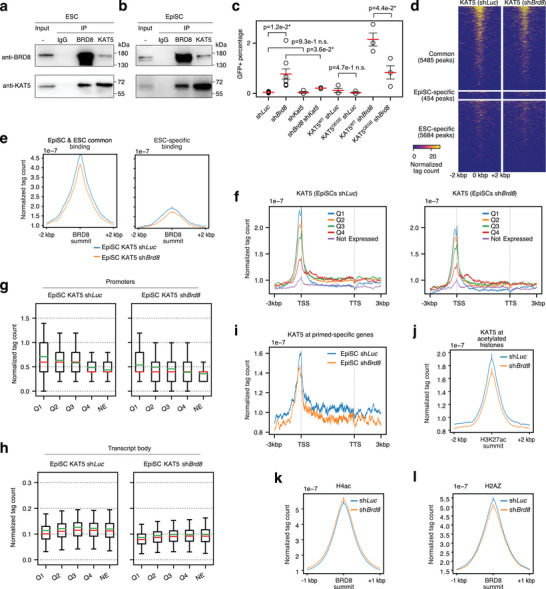
BRD8 requires KAT5 for activity. a) Western blot of Co‐IP with the indicated antibodies (left side) and immunoprecipitations (IPs) in ESCs. The input is shown for comparison. b) Western blot of Co‐IP with the indicated antibodies and immunoprecipitations (IPs) in EpiSCs. The input is shown for comparison. c) GFP+ cell counts on day 6 as counted by FACS in cells transfected with the indicated shRNAs targeting *Brd8*, *Kat5*, or *Luc* as a control, or with a *Kat5* overexpression vector. WT = wildtype, QEGE = Q377E/G380E KAT5 catalytic mutant. GFP+ cells counted by FACS at day 6 of a primed‐to‐naïve‐transition. This experiment was performed in n = 3 or n = 6. The red bar indicates the mean and the error bars are the standard error of the mean. Significance is from a two‐sided Welch's t‐test, * indicates p‐value less than 0.05. d) Heatmap pileups for CUT&Tag of KAT5 in EpiSCs transfected with a control sh*Luc* or sh*Brd8*. Peaks were divided as in Figure 4a by BRD8 binding. The heatmap is centered on BRD8 binding and extends 2 kbp either side of the peak summit. e) Density pileups for KAT5 at BRD8 binding sites in EpiSCs and ESCS (as defined in Figure 4a). The pileups are centered on the BRD8 binding summit. f) A pileup of KAT5 CUT&Tag signals across all genes in the mouse genome. Genes were divided into expressed (normalized tag count >100) and not expressed. The expressed genes were further subdivided into their expression quartiles, Q1‐Q4. The windows of binding are centered on the TSS and TTS (transcription termination site) and all transcripts are scaled to a uniform length. The flanking 5′ and 3′ regions 3 kbp from the TSS or TTS are shown. g) Box plots showing the normalized tag count for KAT5 in EpiSCs transfected with an shRNA targeting *Luc* or *Kat5*, at the promoter (± 500 bp around the TSS). Genes were divided into expressed (normalized tag count >100) and not expressed. The expressed genes were further subdivided into their expression quartiles, Q1‐Q4. The green line is the mean, the red middle line is the median. h) As in panel g, but for the transcript bodies, defined as 500 bp 3′ of the TSS and 500 bp 5′ of the transcription end site (TES). i) A pileup of KAT5 C&T signals across primed‐specific genes (As defined in Figure S3a,b, Supporting Information), in cells transfected with an shRNA targeting *Luc* or *Brd8*. j) Pileup of KAT5 binding in cells transfected with an shRNA targeting *Luc* or *Brd8* at all acetylated histones in EpiSCs. k) Pileup of H4ac in cells transfected with an shRNA targeting *Luc* or *Brd8* at all BRD8 summits in EpiSCs. l) Pileup of H2AZ in cells transfected with an shRNA targeting *Luc* or *Brd8* at all BRD8 summits in EpiSCs.

We next sought to unravel a mechanism to explain how BRD8 drives the reduction of histone acetylation at primed‐specific genes. In our Co‐IP/MS data, BRD8 is associated with components of the NuA4 complex in EpiSCs (Figure 4e). In our Co‐IP/MS data, we could not readily identify the acetyltransferase responsible. We surmised that BRD8 might collaborate with KAT5 (TIP60), which is the main catalytic subunit of the NuA4 complex.^[^ ^35^ ^]^ We employed a Co‐IP Western blot and a Kozak sequence‐driven over‐expression system to test whether BRD8 and KAT5 could interact (Figure S8a, Supporting Information). In this system BRD8 could co‐precipitate KAT5 (Figure S8b, Supporting Information), suggesting that KAT5 may be the histone acetyltransferase critical for BRD8 function. We confirmed the interaction between BRD8 and KAT5 using endogenous proteins in both ESCs and EpiSCs (**Figure** **7**a,b).

We speculated that BRD8 is responsible for correctly localizing KAT5 to primed‐specific genes, and reduced *Brd8* would lead to incorrect localization and a consequent reduction in histone acetylation. Hence, if KAT5 were required for the effect of reduced *Brd8* on the primed‐to‐naïve conversion then overexpression of *Kat5* would synergize with a *Brd8* knockdown. This was indeed the case. When *Kat5* alone was overexpressed in a primed‐to‐naïve conversion it had only a modest effect in improving the percentage of resulting GFP+ naïve cells (Figure 7c). However, the combination of *Kat5* overexpression and *Brd8* knockdown substantially improved the conversion of primed EpiSCs to naïve ESCs (Figure 7c). Importantly, this effect required a catalytically active KAT5 as catalytically inactive KAT5^Q377E/G380E^ (KAT5^QEGE[^ ^61^ ^]^) was incapable of synergizing with the *Brd8* knockdown (Figure 7c). Interestingly, this effect is converse to KAT5's role in ESCs, where it promotes self‐renewal and represses differentiation genes by a mechanism that does not require lysine acetylation activity.^[^ ^62^ ^]^ Potentially mutation of KAT5 would disrupt the interaction between BRD8 and KAT5, however, the two proteins still interacted (Figure S8a, Supporting Information), and the subcellular localization was not altered for the KAT5^QEGE^ mutant. (Figure S8b, Supporting Information). Finally, if KAT5 is required to synergize with BRD8, then a double knockdown of *Brd8* and *Kat5* should no longer increase GFP+ cells. This was also the case, as *Kat5* knockdown alone did not improve GFP+ cell percentages, and a double knockdown reverted the percentage of GFP+ cells to near the control sh*Luc* transfected cells (Figure 7c). These results indicate that functional histone acetyltransferase activity is required for BRD8 function.

We next explored the genome binding of KAT5. CUT&Tag of KAT5 in EpiSCs showed that KAT5 was primarily associated with BRD8‐bound loci in EpiSCs (Figure 7d,e). Importantly, the knockdown of *Brd8* reduced the binding of KAT5, indicating that BRD8 is indeed responsible for anchoring KAT5 to the genome (Figure 7d,e). We next looked at the association of KAT5 binding with gene expression levels. KAT5 binding tended to be higher at the TSSs of expressed genes in Q1 (Figure 7f,g). However, KAT5 binding in the transcript body tended to be higher in Q2‐Q4 genes (Figure 7f,h). Knockdown of *Brd8* reduced KAT5 binding at all gene expression quartiles in the promoter (Figure 7f,g). Based on these data we proposed that KAT5 maintains chromatin at primed‐specific genes. When we measured the level of KAT5 at primed‐specific genes it was indeed high (Figure 7i). Importantly, when *Brd8* was knocked down KAT5 binding was also reduced at primed‐specific genes (Figure 7i). Indicating that BRD8 is anchoring KAT5 to both the TSS and transcribed body of primed‐specific genes. We measured KAT5 binding at acetylated histones, and it was reduced when *Brd8* was knocked down (Figure 7j).

KAT5 has been reported to acetylate H4 histones.^[^ ^63^, ^64^ ^]^ We measured the levels of H4ac by western blot, however there was not much change when *Brd8* was knocked down (Figure 5a). We suspected that changes in H4ac may be context‐specific, hence we performed CUT&Tag for H4ac in EpiSCs transfected with shRNAs targeting *Luc* or *Brd8*. Surprisingly, in the CUT&Tag data H4ac levels were moderately increased upon *Brd8* knockdown (Figure 7k). The effect was subtle though, as at primed‐specific genes the level of H4ac did not change at the TSS, although there was a small increase in the transcript body (Figure S8d, Supporting Information). This was surprising, because KAT5 acetylates histone H4.^[^ ^63^, ^64^ ^]^ We wondered if KAT5 was associated with H4ac loci, and this was indeed the case, and KAT5 binding declined at H4ac loci when *Brd8* was knocked down (Figure S8e, Supporting Information). Our speculation for these conflicting results is that KAT5 is not the main H4 acetyltransferase in EpiSCs. Indeed, there are several other H4ac acetyltransferases expressed at high levels in EpiSCs (Figure S8f, Supporting Information). Additionally, KAT5 also catalyzes acetylation of other histones, including H2A and to a lesser extent H3.^[^ ^61^, ^63^, ^64^, ^65^ ^]^ To explain this discrepancy, we looked at H2AZ as it was previously reported H2AZ decreased when KAT5 was knocked down^[^ ^65^ ^]^ and our epigenetic phenotype was reminiscent of a pattern seen in a *Chd4* knockdown experiment, where H2AZ levels declined.^[^ ^66^ ^]^ Hence, we performed CUT&Tag for H2AZ, and when *Brd8* was knocked down H2AZ was reduced at BRD8‐binding sites (Figure 7l). Overall, these data indicate that BRD8 is responsible for anchoring KAT5 at primed‐specific genes and acetylated histones. Reduced *Brd8* expression leads to disruption of the maintenance of acetylation at primed‐specific genes, which ultimately leads to increased cell plasticity due to the erosion of epigenetic marks.

## 3 Discussion

Our data suggests that BRD8 has dual roles in primed EpiSCs. First, it maintains acetylation at primed‐specific genes to stabilizes cell type. Second, it primes the chromatin accessibility of naive‐specific genes, making them ready for activation. This is accompanied by reduced repressive marks at naïve‐specific genes. Consequently, when *Brd8* expression is reduced EpiSCs become more permissive to the primed‐to‐naïve conversion. The mechanism appears to be caused by a weakening of active marks of chromatin at cell type‐specific genes. This then leads to weak expression of alternative lineage genes (in this case primed‐specific genes) that can result in enhanced cell type conversion. This work highlights how transcriptional activators are important for cell type stability.

BRD8 is a member of the NuA4 epigenetic complex, but surprisingly little is known about BRD8's role. The best characterized biological roles for BRD8 are in cancer, where it has complex roles in both promoting and suppressing tumorigenesis.^[^ ^58^, ^67^, ^68^, ^69^, ^70^, ^71^, ^72^ ^]^ The *BRD8* gene can be translocated to form a BRD8‐PHF1 fusion protein that likely leads to misallocated histone acetylation and presumably gene activation by analogy to an EPC1‐PHF1 fusion that disrupts NuA4.^[^ ^60^ ^]^ Genome wide mapping of BRD8 binding in cancers suggests BRD8 binds to a wide range of cell cycle, DNA replication and immune response genes.^[^ ^69^, ^71^ ^]^ Further, BRD8 may modulate an antagonistic relationship between NuA4 and the co‐repressor complex NuRD via TWIST1 binding,^[^ ^34^ ^]^ suggesting BRD8 mediates a delicate epigenetic balance in normal tissues that is disrupted in cancerous cells. BRD8 has a key role in maintaining *TP53* (p53) expression in glioblastoma.^[^ ^58^ ^]^ Interestingly it represses p53‐target genes by blocking p53 binding to the genome through maintaining compact chromatin. This is opposite to the pattern seen here, where reduced *Brd8* leads to reduced histone acetylation and reduced histone methylation, although the latter change appears indirect. BRD8 can also bind non‐histone proteins, such as the acetylated from of the N‐terminus of TWIST1 to recruit the rest of NuA4 to promote the epithelial‐mesenchymal transition and metastasis.^[^ ^34^ ^]^ Mutations in BRD8 are relatively rare, and are sporadically distributed across many different cancer types instead of being concentrated in a single cancer,^[^ ^68^ ^]^ suggesting BRD8 is playing an accessory role in tumorigenesis. In non‐cancerous tissues BRD8 is involved in adipogenesis, where it modulates H2AZ incorporation.^[^ ^73^ ^]^ Additionally, the accurate splicing of the *Brd8* transcript is required for correct post‐germinal vesicle oocyte development.^[^ ^74^ ^]^ However, beyond those studies, roles for BRD8 in normal biological processes has not been described.

Several epigenetic pathways can be inhibited or promoted to increase the efficiency of the primed‐to‐naïve conversion. Inhibition of the H3K4 methylase KMT2A (MLL1) using a small molecule can promote the primed‐to‐naïve conversion.^[^ ^54^ ^]^ Indeed, we show that reduced *Brd8* expression led to reduced H3K4me1 at BRD8‐bound loci, and also at the TSSs of primed‐specific genes. Interestingly, BRD8 was bound to the promoter of *Kmt2a* in both ESCs and EpiSCs. However, the expression of *Kmt2a* was unaffected in the *Brd8* knockdowns and there was no overlap between genome‐wide binding of BRD8 and KMT2A, suggesting that BRD8 and KMT2A affect the primed‐to‐naïve conversion independently. The transcriptional repressor *Sin3a* when overexpressed promotes the primed‐to‐naïve conversion,^[^ ^75^ ^]^ as does overexpression of the histone H3K27 demethylase *Kdm6b* (JMJD3).^[^ ^55^ ^]^ Inhibition of the H3K79 methyltransferase DOT1L leads to a primed‐to‐naïve conversion with high efficiency.^[^ ^20^ ^]^ The TF *Zfp281* has been identified as a key primed transcriptional regulator that regulates *Tet1*, and thus DNA demethylation, to promote the primed state.^[^ ^24^, ^76^ ^]^ ZFP281 also regulates EHMT1 and its catalytic target H3K9me to control ZIC2 genome‐wide binding.^[^ ^19^ ^]^ There was a modest overlap of BRD8 binding with ZFP281 in EpiSCs, and this may partly explain the changes we see in H3K9me3 levels. Ultimately, these data all point to the idea that several epigenetic barriers are erected that impair the primed‐to‐naïve conversion.

Enhancer loosening was seen previously in the primed to naïve‐transition.^[^ ^45^ ^]^ Potentially, the phenomenon we are seeing here is related to this process, as when *Brd8* was reduced we indeed saw a series of changes in chromatin accessibility at both primed and naïve genes, and a consequent change in enhancer chromatin marks (H3K27ac, H3K4me1). This was reflected in reduced KAT5 binding at primed‐specific genes and at acetylated histones. One interesting discrepancy in our study involves the histone acetylation target of KAT5. When we performed CUT&Tag for H4ac when *Brd8* was knocked down the signal increased. KAT5 acetylates histone H4,^[^ ^63^, ^64^ ^]^ but it has also been reported to acetylate H2A and to a lesser extent H3.^[^ ^61^, ^63^, ^64^, ^65^ ^]^ We speculate that KAT5 is not the major H4 acetyltransferase in EpiSCs, and instead has a role in regulating H2AZ incorporation dynamics, similar to previous reports that demonstrated how KAT5 and BRD8 are linked to H2AZ dynamics.^[^ ^65^, ^73^ ^]^ Overall, these observations suggest enhancers are indeed weakened in response to reduced expression of BRD8.

In the Co‐IP/MS data BRD8 precipitated MORF4L1/2 and MRGBP in ESCs, but not the wider NuA4 complex proteins. This is potentially explained by analogy to the “TINTIN” (Trimer Independent of NuA4 for Transcription Interactions with Nucleosomes) complex seen in.^[^ ^56^ ^]^ In that paper p400NL competes with the rest of the NuA4 complex to bind to BRD8, MORF4L1/2 and MRGBP. Although the full structural details of the NuA4 complex remain to be elucidated,^[^ ^77^ ^]^ and the location of BRD8 remains ambiguous, it appears BRD8 is a peripheral part of the NuA4 complex that associates with the larger NuA4 complex through KAT5 and MORF4L1/2 or MRGBP, although the interaction does not require MORF4L1/2. Overall, our data supports a model for BRD8 in maintaining cell‐type stability. BRD8 helps anchor the NuA4 complex to acetylated histones where it serves a role in maintaining active chromatin at promoters and transcribed gene bodies. Reduced *Brd8* expression disrupts KAT5 binding, leading to disrupted acetylation maintenance, and a consequent weakening of promoters and enhancers. This process occurs indiscriminately at both naive and primed‐specific genes, but if the cells are exposed to a culture environment that favors conversion to a naïve state then the conversion is improved. Altogether, our data shows that BRD8 acts as a barrier for the conversion of the primed cells to the naïve state by helping to maintain a stable cell type through the maintenance of chromatin marks at key promoters.

## 4 Experimental Section

### ESC and EpiSC Cell Culture and the Conversion between the Two Cell Types

Mouse EpiSCs were a kind gift of Chen Jiekai (Guangzhou Institutes of Biomedicine and Health). Briefly, the cells were derived from embryonic day 5.5 mouse embryos generated by mating homozygous Oct4‐GFP transgenic‐allele‐carrying mice (CBA/CaJ × C57BL/6J) with 129/Sv female mice. EpiSCs were cultured feeder‐free on dishes coated with fetal bovine serum (FBS; NATOCOR) in FA medium (N2B27 medium, 15 ng ml^−1^ bFGF (PeproTech) and 20 ng ml^−1^ activin A (PeproTech). N2B27‐based medium: DMEM/F12 (HyClone) and Neurobasal (Gibco) mixed 1:1, supplemented with N2 (Gibco), B27 (Gibco), non‐essential amino acids (Gibco), GlutaMAX (Gibco), sodium pyruvate (Cellgro), penicillin/streptomycin (HyClone), 0.1 mM β‐mercaptoethanol (Gibco). Mouse EpiSCs were passaged using Accutase (Sigma) and seeded as single cells at ≈50 000 cells in a well of a 6‐well plate every 3 days. The medium was changed daily.

Mouse ESCs were maintained feeder‐free on 0.1% gelatin in N2B27 medium supplemented with 2i/LIF at the following final concentrations: 1 µM PD0325901 (Sigma), 3 µM CHIR99021 (Sigma) and 1000 U ml^−1^ leukemia inhibitory factor (LIF; Millipore). Mouse ESCs were passed by 0.05% trypsin‐EDTA (Gibco) dissociation every 3 days.

To reprogram EpiSCs into rESCs, EpiSCs were dissociated into single cells using Accutase and plated at a density of 5000 cells well^−1^ on a 6‐well plate coated with feeder in N2B27+FA medium supplemented with 5 µM Y27632 (Selleck). The next day, N2B27+FA medium was changed into N2B27‐2iL+Vc medium for 5 days, Vc (A4034, Sigma) was used at 50 µg ml^−1^. The medium was changed every day.

### Cell Transfection and shRNAs used in This Study

Lentiviruses were generated from HEK293T cells using the jetPEI (Polyplus transfection). Mouse EpiSCs were infected with the individual lentiviral supernatant with the addition of Polybrene (Sigma) for 8 h followed by selection with puromycin (Selleck) for 2 days before reprogramming. shRNA inserts were cloned into pLKO lentiviral vectors. shRNA target sequences are: shBrd1: 5′‐CTAGAAGCTCAAGGGTATAAA‐3′, shBrd2: 5′‐TTATGTTCTCCAACTGCTAT‐3′, shBrd3: 5′‐GTATGCAGGACTTCAACACCAT‐3′, shBrd9: 5′‐TGGACCTGAGTTCACTGTCTA‐3′, shBrdt: 5′‐GCCAAGTCGACAAACAGCTATT‐3′, shBrd8‐1: 5′‐GGTTCTTCCCATGATACATGG‐3′, shBrd8‐2: 5′‐GGAGTTGGTCCAGTTCCAAGT‐3′, shBrd8‐3: 5′‐GGAAGAGGATCAAGGAGAAGG‐3′, shBrd8‐5: 5′‐GATAGACATCATGGTCTGAGC‐3′, shBrd8‐10: 5′‐GCTGAGATAGTAGCTGGAGTT‐3′, shKat5: 5′‐ACGGAAGCGGAAATCTAATTG‐3′, shMorf4l1‐1: 5′‐TAGTCCTTCTCTTGTACAAAT‐3′, shMorf4l1‐2: 5′‐GTTGCCATAAAGGACAAACAA‐3′, shMorf4l1‐3: 5′‐CCTTGCTTTATTACTGAACTA‐3′, shMorf4l2‐1: 5′‐CCTGAGATTATTCGTGAGAAT‐3′, shMorf4l2: 5′‐CGTGGACAACAATCTGCTGA‐3′, mBrd1‐F1: 5′‐AACACTGACCTACGCACAAGC‐3′, mBrd1‐R1: 5′‐GCCTCTCGCTGTTCTCCTTATT‐3′, mBrd2‐F1: 5′‐ATGCTGCAAAACGTGACTCC‐3′, mBrd2‐R1: 5′‐AAGCTGGTACAGAAGCCATTG‐3′, mBrd3‐F1: 5′‐AAAAAGGCTCCCACCAAGAAG‐3′, mBrd3‐R1: 5′‐TGTCAAGGCTAAGTTGTCGCT‐3′, mBrd8‐F1: 5′‐TCCTTCCTTCACTACTGTTGCC‐3′, mBrd8‐R1: 5′‐ACAGCTTCCAGGGGTACACA‐3′, mBrd9‐F1: 5′‐TTGGAGATGGAAGTCTGCTCT‐3′, mBrd9‐R1: 5′‐GCAACTTGCTAGACAGTGAACT‐3′, mBrdt‐F1: 5′‐AGTGGGCGGTTGACGAATC‐3′, mBrdt‐R1: 5′‐AGTCAGGCAGCTTTAGTTTCAC‐3′.

### Co‐Immunoprecipitation and Mass Spectrometry

Co‐immunoprecipitation mass spectrometry (Co‐IP/MS) was performed as previously described,^[^ ^39^ ^]^ Briefly, proteins were extracted using NP‐40 lysis buffer (50 mM Tris‐HCl pH 7.5, 150 mM NaCl, 0.5% Nonidet P‐40, 1 mM EDTA). Antibodies were pre‐bound to 40 µl Dynabeads protein G or 40 µl protein A (10001D and 10003D, Life Technologies) for 4 °C for 3–5 h. Antibodies used include 10 µg anti‐BRD8 (A300‐220A, Bethyl Laboratories). Dynabeads‐antibody were mixed with proteins and rotated overnight at 4 °C, then the protein‐antibody‐Dynabeads were washed three times with lysis buffer. Proteins were detected on an Agilent 7700X.

Mass spec data was analyzed using MAXQUANT.^[^ ^78^ ^]^ The resulting peptide matches were filtered using glbase3,^[^ ^79^ ^]^ using default filter settings for mouse proteins. Briefly, peptides detected by MAXQUANT were filtered based on several criteria. A protein was considered detected if it had at least 1 unique peptide and a minimum intensity of 1 000 000 (Razor+Unique). A protein was considered an interactor with BRD8 if the intensity was at least 2‐fold above the anti‐FLAG control. The resulting peptides were filtered to remove a list of common contaminating proteins (Briefly, protein names starting with Rpl, Rps, Tub, Gapdh, Act, Myh, Ighg, Iglv, Col, Golga, Kif, Myl, Krt, Eif, Vim, Atp, Igkv, Ighv). The resulting table of filtered proteins was in Table S1 (Supporting Information). In some figures, the proteins were selected and filtered based on their presence in the epigenetic factor or transcription factor databases EpiFactors and AnimalTFDB.^[^ ^32^, ^80^ ^]^

### Flow Cytometry

Cells were dissociated into single cells using Accutase and collected using centrifugation. After washing once with PBS, the cell pellet was resuspended with PBS containing 0.1% BSA, followed by filtration using a cell strainer (BD Biosciences) to remove large clumps of cells. The cells were then analyzed using a FACSCanto flow cytometer (BD Biosciences). The GFP fluorescence intensity was detected in the FITC channel. Data were analyzed with FlowJo (v10.8.1) software.

### Western Blot

After being dissociated and counted, 1 × 10^6^ cells were collected and lysed in RIPA buffer (100 µl) (Beyotime, P0013B) with protease inhibitor cocktail (Roche) on ice for 5 min and boiled for 5 min at 100 °C. The samples were separated using 10%–12% SDS–PAGE and transferred onto a polyvinylidene difluoride membrane (Millipore) using a wet transfer system, and then incubated with the primary antibodies and secondary antibodies. The goldband plus 3‐color regular range protein marker (YEASEN, 20350ES) was used to estimate protein size. The following primary antibodies were used: anti‐H3 (Abcam, ab1791, 1:2000), anti‐H3K27ac (Abcam, ab4729, 1:2000), anti‐H3K9me3 (Abcam, ab8898, 1:2000), anti‐H4acetyl (Millipore, 06–866, 1:2000); anti‐BRD8 (Abcam, ab17969, 1:2000), anti‐BRD8 (Bethyl, A300‐220A, 1:2000), anti‐β‐actin (Sigma, A5541, 1:4000), anti‐KAT5 (Proteintech,10827‐1‐AP, 1:1000), anti‐HA (haemagglutinin; Sigma H6908, 1:1000), anti‐HA (YEASEN, 30704ES, 1:1000), anti‐FLAG (Sigma, F1804, 1:5000).

### RNA‐Seq and Analysis

RNA‐seq was performed as previously described.^[^ ^4^, ^47^ ^]^ Briefly, RNA was purified using RNAzol RT (RN190, MRC) according to the manufacturer's instructions. The samples were prepared for sequencing with RNA‐seq NEB Next Ultra RNA Library Prep Kit (7530, NEB). Samples were sequenced on an Illumina Novaseq 6000.

RNA‐seq data was aligned to the mouse genome using STAR,^[^ ^81^ ^]^ using the settings “–outFilterMultimapNmax 100 –winAnchorMultimapNmax 100 –outMultimapperOrder Random –runRNGseed 777 –outSAMmultNmax 1 –outSAMtype BAM Unsorted –twopassMode Basic –outFilterType BySJout –alignSJoverhangMin 8 –alignSJDBoverhangMin 1 –outFilterMismatchNmax 999 –alignIntronMin 20 –alignIntronMax 1 000 000 –alignMatesGapMax 1 000 000”. Counts were assigned to features using scTE/te_counter (https://github.com/oaxiom/te_counter) against transcripts (GENCODE v32) or transposable elements,^[^ ^82^ ^]^ and reads were GC normalized using EDASeq.^[^ ^83^ ^]^ Differentially expressed genes were called using DESeq2.^[^ ^84^ ^]^ Genes were considered differentially regulated if they had a q‐value or 0.01 (Bonferroni‐Hochberg corrected p‐value) and a fold‐change of 2 or 4 (specified in the appropriate figure legend). Gene ontology analysis was performed using GO‐seq,^[^ ^85^ ^]^ and GSEA used fgsea.^[^ ^86^ ^]^ Other analysis was performed using glbase3.^[^ ^79^ ^]^

### RT‐qPCR

Total RNA from cells was isolated using RNAzol RT (MRC, RN190) according to the manufacturer's protocols. cDNA synthesis by using a PrimeScript RT Master Mix (Takara, RR036A). Real‐time PCR was performed in triplicate using SYBR Premix Ex Taq (Takara, RR820A) and using a Biorad Real‐time PCR system. The primers used include: mMorf4l1‐F1: GGACCCTAAGCCGAAATTCCA, mMorf4l1‐R1: TGTTTGTCCTTTATGGCAACCT, mMorf4l2‐F1: CTCAAACTCGTGGACAACAATCT, mMorf4l2‐R1: TCCTGGTCTTCCGCACAGAA, Otx2‐F1: TATCTAAAGCAACCGCCTTACG, Otx2‐R1: GCCCTAGTAAATGTCGTCCTCTC, mT‐F1: AACTGGTCTAGCCTCGGAGT, mT‐R1: AGCAGCCCCTTCATACATCG, mTcp2L1‐F1: AGGTGCTGACCTCCTGAAGA, mTcp2L1‐R1: GTTTTGCTCCAGCTCCTGAC, mNanog‐F1: CTTTCACCTATTAAGGTGCTTGC, mNanog‐R1: TGGCATCGGTTCATCATGGTAC, mVim‐F1: CGGCTGCGAGAGAAATTGC, mVim‐R1: CCACTTTCCGTTCAAGGTCAAG, mPouf5‐F1: TGGATCCTCGAACCTGGCTA, mPouf5‐R1: GGAGGTTCCCTCTGAGTTGC, mSall4‐F1: TCCAACATTTATCCGAGCACAG, mSall4‐R1: TGGCAGACGAGAAGTTCTTTC.

### ATAC‐Seq and CUT&Tag and Their Analysis

ATAC‐seq was performed as described.^[^ ^47^, ^48^ ^]^ Briefly, nuclei from ≈50 000 cells were extracted with lysis buffer (10 mM Tris‐HCl pH 7.4, 10 mM NaCl, 3 mM MgCl_2_, and 0.2% (v/v) IGEPAL CA‐630). Tagmentation reactions were performed in situ by the addition of 50 µl reaction mix from a TruePrep DNA Library Prep Kit (TD502, Vazyme). DNA fragments were purified using the MinElute PCR Purification Kit (28 004, Qiagen). ATAC‐seq libraries were amplified with PCR for an appropriate number of cycles 18 and the sequence index was added by TruePrep Index Kit V2 for Illumina (TD202, Vazyme). The amplified DNA libraries were purified using the VAHTS DNA Clean Beads (N411‐02, Vazyme).

CUT&Tag was performed as described.^[^ ^50^ ^]^ Briefly, 1 × 10^5^ cells were washed twice with wash buffer (20 mM Tris‐HCl pH 7.4, 150 mM NaCl, 0.5 mM spermidine and 1× protease inhibitors). Then cells were bound to concanavalin A beads (BP531, Bangs Laboratories) in binding buffer (20 mM Tris‐HCl pH 7.4, 10 mM KCl, 1 mM CaCl_2_ and 1 mM MnCl_2_). The bead‐bound cells were washed once with buffer containing 0.01% digitonin, and primary antibodies were added to bead‐bound cells in antibody buffer (4 mM EDTA and 0.2% BSA in wash buffer containing 0.01% digitonin) and rotated overnight at 4 °C. The primary antibodies below were used: anti‐BRD8 (ab17969, Abcam), anti‐KAT5 (10827‐1‐AP, Proteintech), anti‐H3K27me3 (07‐449, Millipore), anti‐H3K4me1 (ab176877, Abcam), anti‐H3K4me3 (ab8580, Abcam), anti‐H3K27ac (ab4279, Abcam), anti‐H3K9me3 (ab8898, Abcam), anti‐H4ac (06‐866, Millipore), and anti‐H2AZ (A4599, Abclonal). A secondary antibody (ABIN101961, Antibodies‐Online) was added to bead‐cells‐antibody in wash buffer and further incubated at RT for 1 h. pG‐Tn5 (S603‐02, Vazyme) was added to cells in “300‐wash” buffer (20 mM Tris‐HCl pH 7.4, 300 mM NaCl, 0.5 mM spermidine and 1× protease inhibitors, 0.01% digitonin) at RT for 1 h. Tn5 was activated with 300‐wash buffer supplemented with 10 mM MgCl_2_ and washed five times with 500 µl 300‐wash buffer. To stop the Tn5 reaction, 10 µl of 0.5 M EDTA, 3 µl of 10% SDS and 1.5 µl of proteinase K (20 mg ml^−1^) were added and incubated at 55 °C for 1 h. DNA fragments were extracted and PCR amplified using NEBNext HiFi 2× PCR Master mix (M0541L, NEB) using the settings: 72 °C, 5 min, 98 °C 30 s and 12–14 cycles of 98 °C 10 s, 63 °C 10 s and 72 °C 30 s. 300–500 bp DNA fragments were purified with the VAHTS DNA Clean Beads (N411‐02, Vazyme). Libraries were sequenced by an Illumina sequencer.

For the ChIP‐seq, ATAC‐seq and CUT&Tag, these data contain a large number of Tn5 adaptors which were first trimmed using cutadapt. The data was aligned to the mouse mm10 genome using botwtie2,^[^ ^87^ ^]^ using the options “–very‐sensitive –no‐unal –no‐mixed –no‐discordant”, and for ATAC‐seq only the extra option ‘‐X 2000′ was also used. Aligned reads were filtered to include only correctly paired reads with a quality score > 20 (samtools view ‐F 1804 ‐q 20) that mapped to a standard chromosome. Peaks were detected using MACS2,^[^ ^88^ ^]^ using the mouse genome and default parameters. Peaks analysis was unified using redefine_peaks, as described.^[^ ^49^ ^]^ Epigenetic states were estimated using ChromHMM,^[^ ^59^ ^]^ using custom state models for EpiSCs based on the epigenetic data generated in this study. Transcription factor motifs were detected using HOMER.^[^ ^89^ ^]^ Other analysis was performed using glbase3.^[^ ^79^ ^]^

### Data Availability

Data generated in this study was available under accession number GSE253033. Several datasets were reanalyzed as part of this work. Including ChIP‐seq data from the following studies: OCT4 ESC, SOX2 ESC, KLF4 ESC, TFCP2L1 ESC, ESRRB ESC (all GSE11431),^[^ ^53^ ^]^ PRDM15 ESC (GSE73692),^[^ ^52^ ^]^ OTX2 EpiSC, ZIC2 EpiSC, SOX2 EpiSC, OCT4 EpiSC, OCT6 EpiSC (All GSE74636),^[^ ^10^ ^]^ MLL1 EpiSC (GSE73992),^[^ ^54^ ^]^ and ZFP281 EpiSC (GSE93042).^[^ ^55^ ^]^ RNA‐seq data from the following studies: PRJEB6168,^[^ ^40^ ^]^ GSE56096,^[^ ^41^ ^]^ GSE58733,^[^ ^42^ ^]^ GSE39656,^[^ ^43^ ^]^ PRJEB7132,^[^ ^44^ ^]^ SRR1274703,^[^ ^45^ ^]^ GSE137627.^[^ ^46^ ^]^

### Statistics and Reproducibility

No statistical test was used to determine the sample size. The investigator was not blinded to the experimental details. Differential gene expression was calculated using DESeq2 (v1.36.0). A gene was considered significantly differentially regulated if it had an absolute fold‐change of at least 2 and a Bonferroni‐Hochberg corrected p‐value (q‐value) of <0.01. Gene ontology analysis was performed using goseq (v1.48.0) and statistics were calculated using goseq's internal statistical model. A gene ontology category was considered significantly enriched if there were at least 50 genes in that GO term and a Bonferroni‐Hochberg corrected p‐value (q‐value) of <0.01. GSEA was performed using fgsea (v1.22.0). Gene sets were considered enriched or depleted if they had an absolute NES (normalized enrichment score) of at least 1.5 and a Bonferroni‐Hochberg corrected p‐value (q‐value) of <0.01.

## Conflict of Interest

The authors declare no conflict of interest.

## Author Contributions

L.S. and X.F. contributed equally to this work. L.S. designed the study, performed the majority of experiments, acquired funding, and helped prepare the manuscript; F.X. performed key experiments. G.M., Z.X. and Y.Z. performed experiments. L.S.Y. performed some of the bioinformatic analysis. D.L and R.J contributed ideas and helped prepare the manuscript. A.P.H. designed the study, performed the bioinformatic analysis, acquired funding, supervised the study, and wrote the manuscript. All authors revised the manuscript.

## Supporting information



Supporting Information

Supplemental Table 1

Supplemental Table 2

## Data Availability

The data that support the findings of this study are openly available in GEO at https://www.ncbi.nlm.nih.gov/geo/query/acc.cgi?acc=GSE242351, reference number 253033.
