# Supplementary material for: BRD8 Guards the Pluripotent State by Sensing and Maintaining Histone Acetylation
Source: Adv Sci (Weinh). 2024 Dec 10;12(5):2409160. doi: 10.1002/advs.202409160 (PMC11792058; doi:10.1002/advs.202409160)

ADVANCED SCIENCE
Open Access

Supporting Information

for *Adv. Sci.*, DOI 10.1002/advs.202409160

BRD8 Guards the Pluripotent State by Sensing and Maintaining Histone Acetylation

*Li Sun, Xiuling Fu, Zhen Xiao, Gang Ma, Yibin Zhou, Haoqing Hu, Liyang Shi, Dongwei Li, Ralf Jauch and Andrew Paul Hutchins**

BRD8 guards the pluripotent state by sensing and maintaining histone acetylation

Supplementary Tables

Table S1. Table of naïve and primed-specific genes as defined as differentially expressed genes, with an absolute log2 (fold-change) of > 2.0 and a Bonferroni-Hochberg corrected p-value (q-value) < 0.01.

Table S2. Summary of the Co-IP Mass spectrometry results

References

[1] J. Yang, D. J. Ryan, W. Wang, J. C. Tsang, G. Lan, H. Masaki, X. Gao, L. Antunes, Y. Yu, Z. Zhu, J. Wang, A. A. Kolodziejczyk, L. S. Campos, C. Wang, F. Yang, Z. Zhong, B. Fu, M. A. Eckersley-Maslin, M. Woods, Y. Tanaka, X. Chen, A. C. Wilkinson, J. Bussell, J. White, R. Ramirez-Solis, W. Reik, B. Gottgens, S. A. Teichmann, P. P. L. Tam, H. Nakauchi, X. Zou, L. Lu, P. Liu, *Nature* **2017**, *550* (7676), 393, https://doi.org/10.1038/nature24052.

[2] C. Buecker, R. Srinivasan, Z. Wu, E. Calo, D. Acampora, T. Faial, A. Simeone, M. Tan, T. Swigut, J. Wysocka, *Cell Stem Cell* **2014**, *14* (6), 838, https://doi.org/10.1016/j.stem.2014.04.003.

[3] R. Lowe, C. Gemma, V. K. Rakyan, M. L. Holland, *BMC Genomics* **2015**, *16* (1), 295, https://doi.org/10.1186/s12864-015-1506-4.

[4] C. A. Klattenhoff, J. C. Scheuermann, L. E. Surface, R. K. Bradley, P. A. Fields, M. L. Steinhauser, H. Ding, V. L. Butty, L. Torrey, S. Haas, R. Abo, M. Tabebordbar, R. T. Lee, C. B. Burge, L. A. Boyer, *Cell* **2013**, *152* (3), 570, https://doi.org/10.1016/j.cell.2013.01.003.

[5] Y. Takashima, G. Guo, R. Loos, J. Nichols, G. Ficz, F. Krueger, D. Oxley, F. Santos, J. Clarke, W. Mansfield, W. Reik, P. Bertone, A. Smith, *Cell* **2014**, *158* (6), 1254, https://doi.org/10.1016/j.cell.2014.08.029.

[6] D. C. Factor, O. Corradin, G. E. Zentner, A. Saiakhova, L. Song, J. G. Chenoweth, R. D. McKay, G. E. Crawford, P. C. Scacheri, P. J. Tesar, *Cell Stem Cell* **2014**, *14* (6), 854, https://doi.org/10.1016/j.stem.2014.05.005.

[7] Y. Huang, H. Zhang, L. Wang, C. Tang, X. Qin, X. Wu, M. Pan, Y. Tang, Z. Yang, I. A. Babarinde, R. Lin, G. Ji, Y. Lai, X. Xu, J. Su, X. Wen, T. Satoh, T. Ahmed, V. Malik, C. Ward, G. Volpe, L. Guo, J. Chen, L. Sun, Y. Li, X. Huang, X. Bao, F. Gao, B. Liu, H. Zheng, R. Jauch, L. Lai, G. Pan, J. Chen, G. Testa, S. Akira, J. Hu, D. Pei, A. P. Hutchins, M. A. Esteban, B. Qin, *Nat Commun* **2020**, *11* (1), 5061, https://doi.org/10.1038/s41467-020-18900-z.

[8] A. P. Hutchins, Z. Yang, Y. Li, F. He, X. Fu, X. Wang, D. Li, K. Liu, J. He, Y. Wang, J. Chen, M. A. Esteban, D. Pei, *Nucleic Acids Res* **2017**, *45* (5), 2354, https://doi.org/10.1093/nar/gkx054.

[9] X. Chen, H. Xu, P. Yuan, F. Fang, M. Huss, V. B. Vega, E. Wong, Y. L. Orlov, W. Zhang, J. Jiang, Y. H. Loh, H. C. Yeo, Z. X. Yeo, V. Narang, K. R. Govindarajan, B. Leong, A. Shahab, Y. Ruan, G. Bourque, W. K. Sung, N. D. Clarke, C. L. Wei, H. H. Ng, *Cell* **2008**, *133* (6), 1106, https://doi.org/10.1016/j.cell.2008.04.043.

[10] S. Mzoughi, J. Zhang, D. Hequet, S. X. Teo, H. Fang, Q. R. Xing, M. Bezzi, M. K. Y. Seah, S. L. M. Ong, E. M. Shin, H. Wollmann, E. S. M. Wong, M. Al-Haddawi, C. L. Stewart, V.

Tergaonkar, Y. H. Loh, N. R. Dunn, D. M. Messerschmidt, E. Guccione, *Nat Genet* **2017**, *49* (9), 1354, https://doi.org/10.1038/ng.3922.

[11] K. Matsuda, T. Mikami, S. Oki, H. Iida, M. Andrabi, J. M. Boss, K. Yamaguchi, S. Shigenobu, H. Kondoh, *Development* **2017**, *144* (11), 1948, https://doi.org/10.1242/dev.143479.

[12] H. Zhang, S. Gayen, J. Xiong, B. Zhou, A. K. Shanmugam, Y. Sun, H. Karatas, L. Liu, R. C. Rao, S. Wang, A. I. Nesvizhskii, S. Kalantry, Y. Dou, *Cell Stem Cell* **2016**, *18* (4), 481, https://doi.org/10.1016/j.stem.2016.02.004.

[13] X. Huang, S. Balmer, F. Yang, M. Fidalgo, D. Li, D. Guallar, A. K. Hadjantonakis, J. Wang, *Elife* **2017**, *6*, https://doi.org/10.7554/eLife.33333.

[14] T. Ikura, V. V. Ogryzko, M. Grigoriev, R. Groisman, J. Wang, M. Horikoshi, R. Scully, J. Qin, Y. Nakatani, *Cell* **2000**, *102* (4), 463, https://doi.org/10.1016/s0092-8674(00)00051-9.

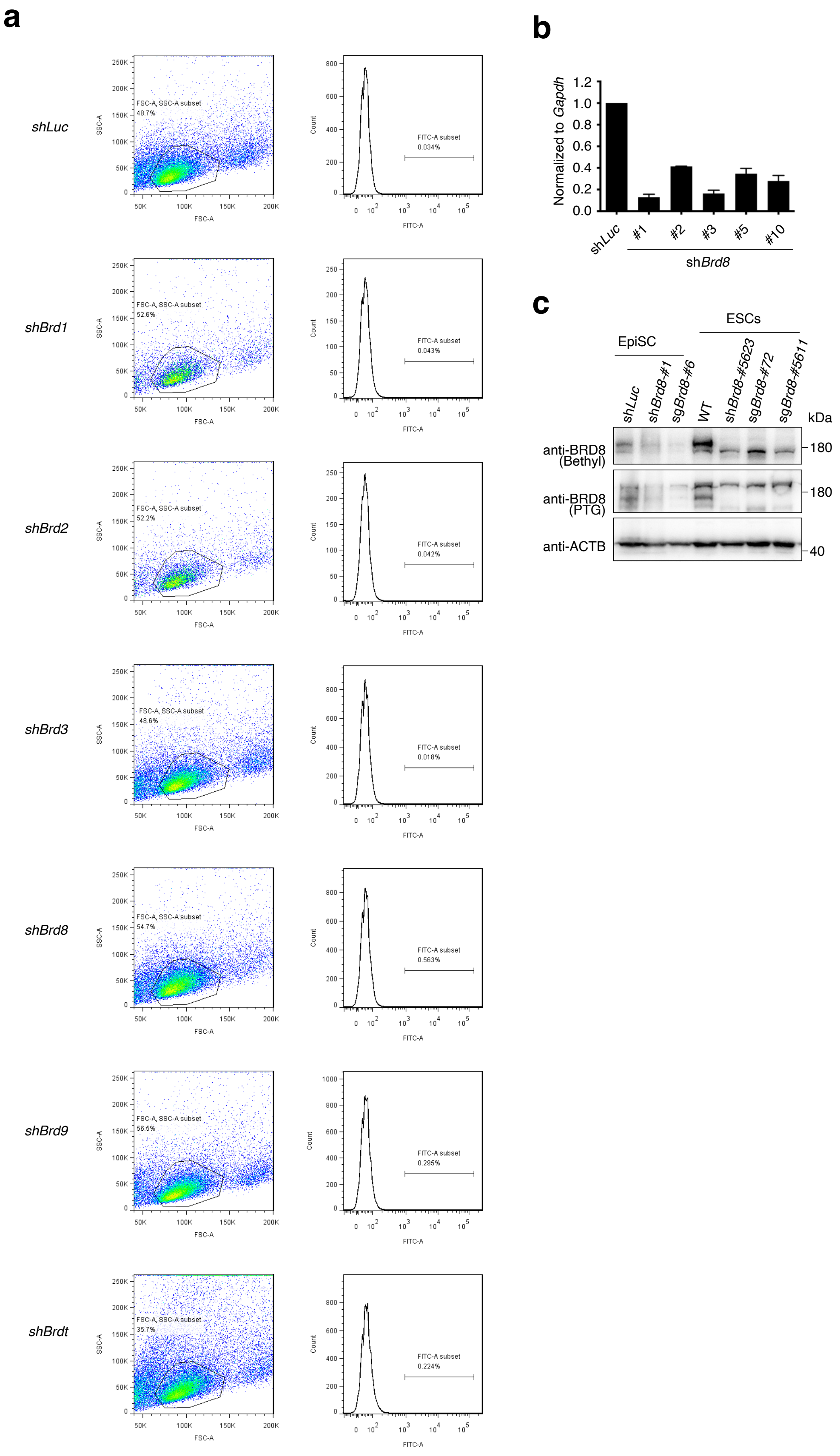

a
shLuc
shBrd1
shBrd2
shBrd3
shBrd8
shBrd9
shBrdt
FSC-A, SSC-A subset
48.7%
52.6%
52.2%
48.6%
54.7%
56.5%
35.7%
FITC-A subset
0.034%
0.043%
0.042%
0.018%
0.563%
0.295%
0.224%
SSC-A
FSC-A
Count
FITC-A
b
Normalized to Gapdh
shLuc
#1
#2
#3
#5
#10
shBrd8
c
EpiSC
ESCs
shLuc
shBrd8-#1
sgBrd8-#6
WT
shBrd8-#5623
sgBrd8-#72
sgBrd8-#5611
kDa
anti-BRD8 (Bethyl)
anti-BRD8 (PTG)
anti-ACTB
180
180
40

Figure S1

Figure S1. BRD proteins impair the primed-to-naïve transition.

a Example flow cytometry histograms for EpiSCs at day 6 of the primed-to-naïve transition transfected with the indicated shRNAs. The left plots show the forward scatter (FSC) and the side scatter (SSC) and the gating strategy used. The right plots show the GFP+ histogram and gating used to define percent positive.

b Bar charts showing RT-qPCR for *Brd8* in EpiSCs transfected with shRNAs targeting *Brd8*. 5 different shRNAs were used. Data is normalized to *Gapdh* expression. The experiment was repeated three times. Error bars are standard error of the means.

c Western blot of EpiSCs or ESCs for BRD8 using two antibodies (PTG and Bethyl) in cells transfected with the indicated shRNA. sgRNAs were also used to show the location of the BRD8 band. BRD8 is the band just below the 180 kDa marker.

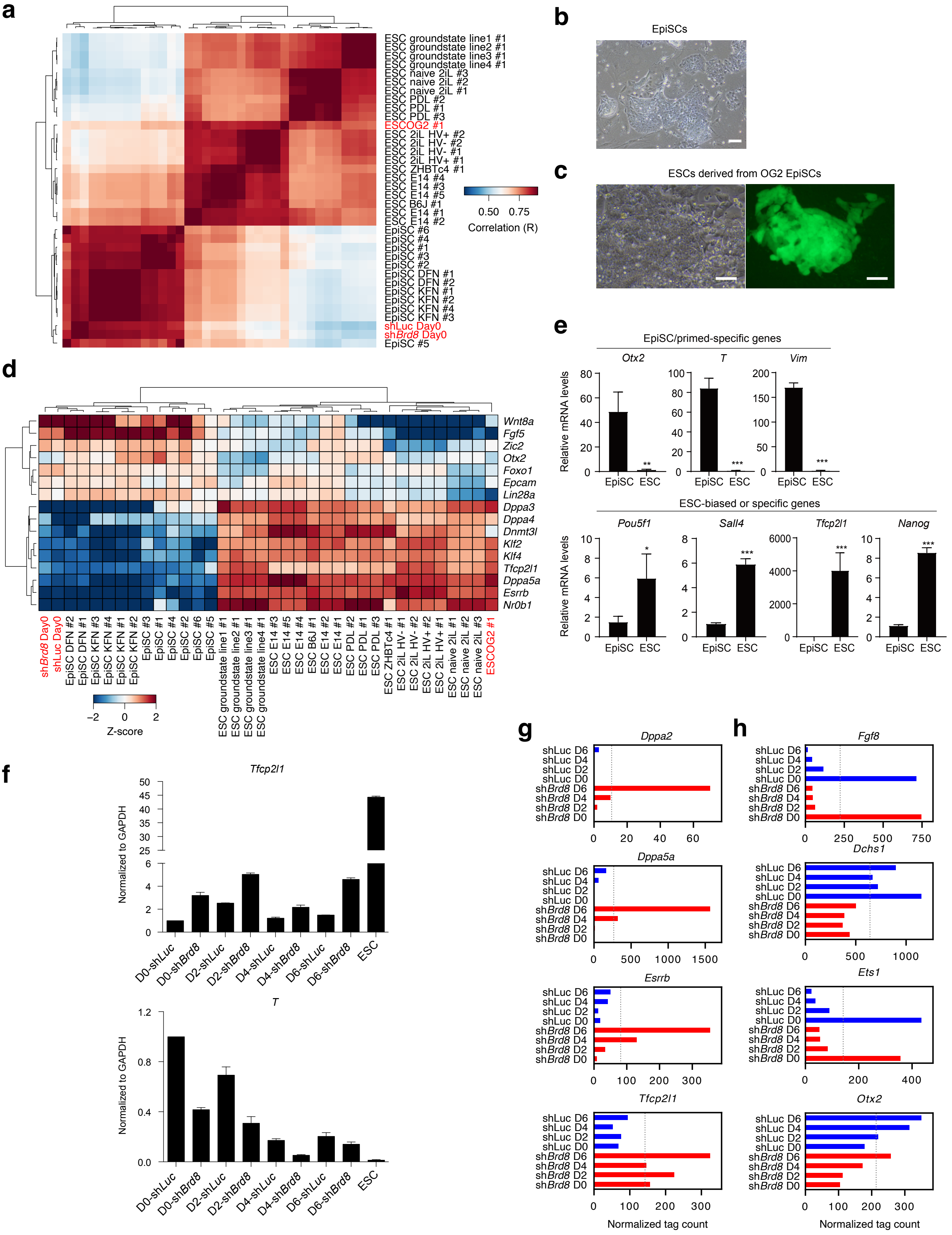
a
ESC groundstate line1 #1
ESC groundstate line2 #1
ESC groundstate line3 #1
ESC groundstate line4 #1
ESC naive 2iL #3
ESC naive 2iL #2
ESC naive 2iL #1
ESC PDL #2
ESC PDL #1
ESC PDL #3
ESCOG2 #1
ESC 2iL HV+ #2
ESC 2iL HV- #2
ESC 2iL HV- #1
ESC 2iL HV+ #1
ESC ZHBTc4 #1
ESC E14 #4
ESC E14 #3
ESC E14 #5
ESC B6J #1
ESC E14 #1
ESC E14 #2
EpiSC #6
EpiSC #4
EpiSC #1
EpiSC #3
EpiSC #2
EpiSC DFN #1
EpiSC DFN #2
EpiSC KFN #1
EpiSC KFN #2
EpiSC KFN #4
EpiSC KFN #3
shLuc Day0
shBrd8 Day0
EpiSC #5
0.50 0.75
Correlation (R)
b
EpiSCs
c
ESCs derived from OG2 EpiSCs
d
Wnt8a
Fgf5
Zic2
Otx2
Foxo1
Epcam
Lin28a
Dppa3
Dppa4
Dnmt3l
Klf2
Klf4
Tfcp2l1
Dppa5a
Esrrb
Nr0b1
–2 0 2
Z-score
e
EpiSC/primed-specific genes
Otx2
T
Vim
Relative mRNA levels
EpiSC ESC
ESC-biased or specific genes
Pou5f1
Sall4
Tfcp2l1
Nanog
f
Tfcp2l1
Normalized to GAPDH
D0-shLuc
D0-shBrd8
D2-shLuc
D2-shBrd8
D4-shLuc
D4-shBrd8
D6-shLuc
D6-shBrd8
ESC
T
g
Dppa2
Dppa5a
Esrrb
Tfcp2l1
shLuc D6
shLuc D4
shLuc D2
shLuc D0
shBrd8 D6
shBrd8 D4
shBrd8 D2
shBrd8 D0
Normalized tag count
h
Fgf8
Dchs1
Ets1
Otx2
Normalized tag count

Figure S2

Figure S2. Confirmation of the OG2 EpiSC and ESC lines used in this study.

- **a** Co-correlation of the ESC and EpiSC lines used in this study (marked in red), versus RNA-seq data for ESCs and EpiSCs from other researchers. Co-correlation (R^2) heatmap for a selection of mouse ESC and EpiSC lines. RNA-seq data was reanalyzed from: PRJEB6168 [1], GSE56096 [2], GSE58733 [3], GSE39656 [4], PRJEB7132 [5], SRR1274703 [6], GSE137627 [7]. Naïve cell types were defined as those grown in serum+LIF (SL) or in 2iL, whilst primed cells were defined as EpiSCs.
- **b** Microscope images of EpiSCs derived in this study. Scale bar = 20 µm.
- **c** Fluorescence images of ESCs derived from the generated EpiSCs. The EpiSCs are derived from OG2 mice and contain a *Pou5f1*::GFP reporter that expresses GFP only in ESCs. Scale bar = 20 µm.
- **d** Heatmap of the expression of selected marker genes for mESCs and EpiSCs. Samples used here come from multiple independent studies. RNA-seq data was reanalyzed from: PRJEB6168 [1], GSE56096 [2], GSE58733 [3], GSE39656 [4], PRJEB7132 [5], SRR1274703 [6], GSE137627 [7]. Naïve cell types were defined as those grown in serum+LIF (SL) or in 2iL, whilst primed cells were defined as EpiSCs.
- **e** Bar charts showing RT-qPCR for theESC/Primed-specific genes *Otx2*, *T* and *Vim*, genes that are expressed in both ESCs and EpiSCs, but at aa lower level in EpiSCs, *Pou5f1*, *Sall4*, and *Nanog*, and the naïve-specific gene *Tfcp2l1* in ESCs or EpiSCs generated in this study. The experiment was repeated three times. Error bars are standard error of the means. * indicates significance <0.05, ** < 0.01 *** < 0.001. Significance is from two-sided unpaired Student's t-test.
- **f** Bar charts showing RT-qPCR for a naïve-specific gene (*Tfcp2l1*) and a primed-specific gene (*T*) in a primed-to-naïve transition cells transfected with a control shRNA against *Luc* or against *Brd8*. The experiment was repeated three times. Error bars are standard error of the means.
- **g** Bar charts showing the expression levels of the indicated naïve-specific genes on days 0 to 6 of a primed-to-naïve transition. Cells were transfected with the indicated shRNA.
- **h** Bar charts showing the expression levels of the indicated primed-specific genes on days 0 to 6 of a primed-to-naïve transition. Cells were transfected with the indicated shRNA.

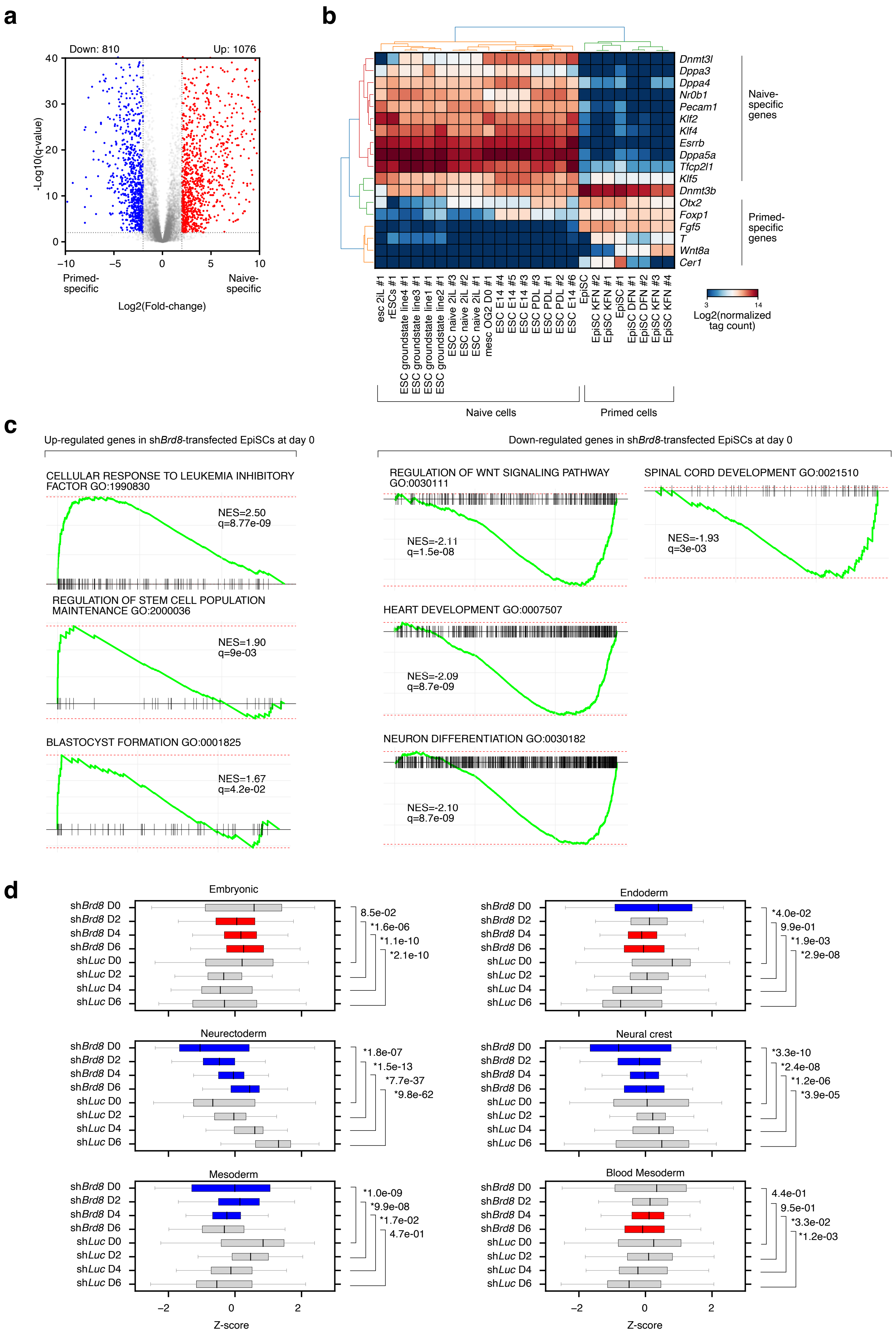
a
Down: 810
Up: 1076
-Log10(q-value)
Primed-specific
Naive-specific
Log2(Fold-change)
b
Dnmt3l
Dppa3
Dppa4
Nr0b1
Pecam1
Klf2
Klf4
Esrrb
Dppa5a
Tfcp2l1
Klf5
Dnmt3b
Otx2
Foxp1
Fgf5
T
Wnt8a
Cer1
Naive-specific genes
Primed-specific genes
Log2(normalized tag count)
Naive cells
Primed cells
c
Up-regulated genes in shBrd8-transfected EpiSCs at day 0
Down-regulated genes in shBrd8-transfected EpiSCs at day 0
CELLULAR RESPONSE TO LEUKEMIA INHIBITORY FACTOR GO:1990830
NES=2.50
q=8.77e-09
REGULATION OF STEM CELL POPULATION MAINTENANCE GO:2000036
NES=1.90
q=9e-03
BLASTOCYST FORMATION GO:0001825
NES=1.67
q=4.2e-02
REGULATION OF WNT SIGNALING PATHWAY GO:0030111
NES=-2.11
q=1.5e-08
SPINAL CORD DEVELOPMENT GO:0021510
NES=-1.93
q=3e-03
HEART DEVELOPMENT GO:0007507
NES=-2.09
q=8.7e-09
NEURON DIFFERENTIATION GO:0030182
NES=-2.10
q=8.7e-09
d
Embryonic
8.5e-02
*1.6e-06
*1.1e-10
*2.1e-10
Endoderm
*4.0e-02
9.9e-01
*1.9e-03
*2.9e-08
Neurectoderm
*1.8e-07
*1.5e-13
*7.7e-37
*9.8e-62
Neural crest
*3.3e-10
*2.4e-08
*1.2e-06
*3.9e-05
Mesoderm
*1.0e-09
*9.9e-08
*1.7e-02
4.7e-01
Blood Mesoderm
4.4e-01
9.5e-01
*3.3e-02
*1.2e-03
shBrd8 D0
shBrd8 D2
shBrd8 D4
shBrd8 D6
shLuc D0
shLuc D2
shLuc D4
shLuc D6
Z-score

Figure S3

Figure S3. Reduced *Brd8* accelerates the primed-to-naïve transition.

a Volcano plot of a selection of EpiSC samples versus a selection of mESC samples. Genes were considered specific to naïve or primed if they had an absolute fold-change of at least 2.0 and a Bonferroni-Hochberg corrected p-value (q-value) of <0.01. RNA-seq data was reanalyzed from: PRJEB6168 [1], GSE56096 [2], GSE58733 [3], GSE39656 [4], PRJEB7132 [5], SRR1274703 [6], GSE137627 [7]. Naïve cell types were defined as those grown in serum+LIF (SL) or in 2iL, whilst primed cells were defined as EpiSCs. See **panel b** for the specific samples.

b Heatmap of a selection of naïve and primed-specific genes (from **panel a**), in the indicated RNA-seq samples as in **panel a**.

c Plots showing the enrichment of selected gene ontology terms for genes significantly up-regulated (left panel) or down-regulated (right panel) at day 0 in sh*Brd8* transfected EpiSCs versus shLuc (control transfected EpiSCs. genes using GSEA. A term was considered significant if it had an absolute normalized enrichment score (NES) of at least 1.5 and a q-value of <0.01.

d Boxplots showing sums of Z-scores for the indicated germ lineage-specific genes as defined in [8]. Significance is from a two-sided Welch's t-test comparing sh*Brd8* versus sh*Luc* for each time point pair. * indicates a p-value < 0.05, red boxes indicate up-regulated, blue indicates significantly down-regulated.

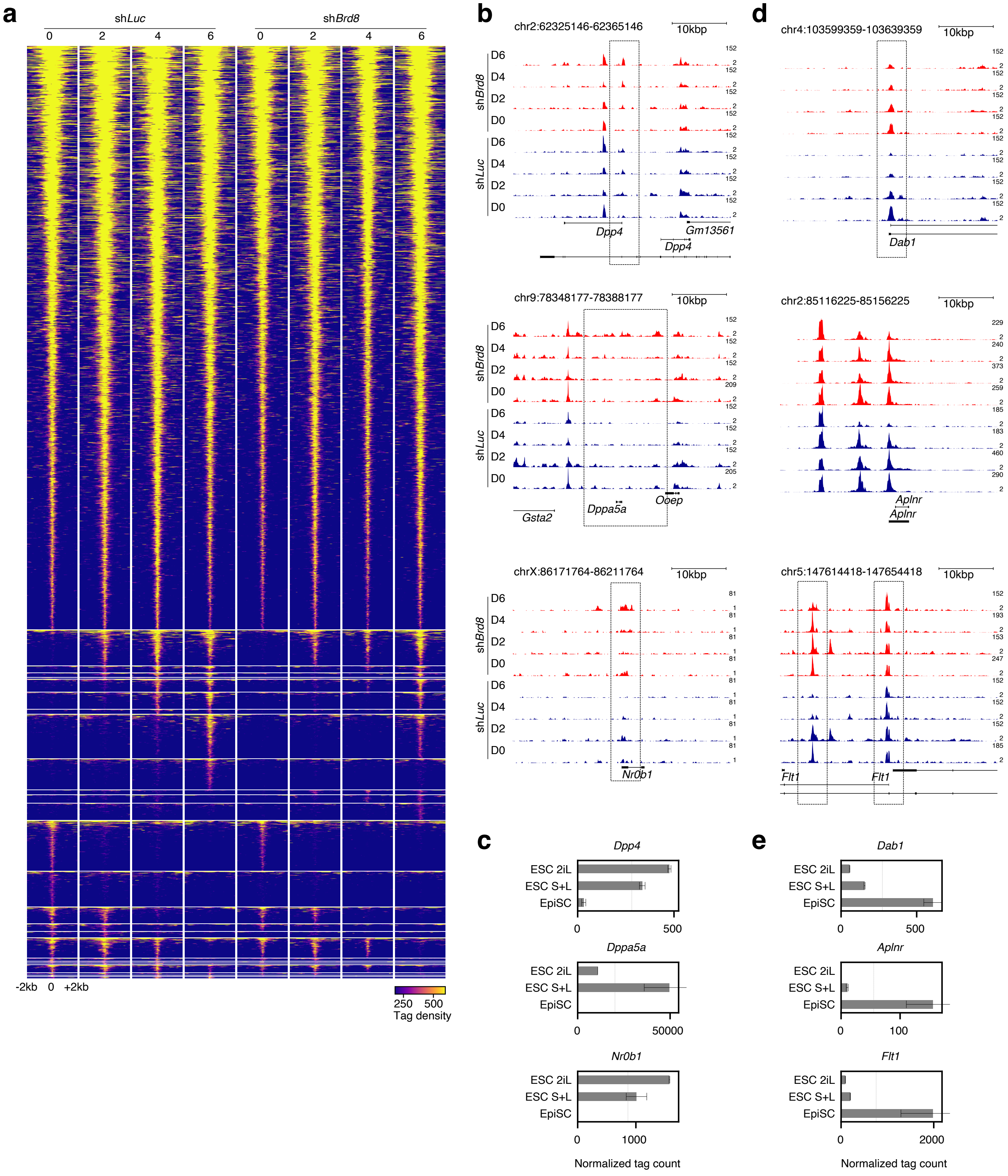
a
shLuc
shBrd8
0
2
4
6
-2kb 0 +2kb
250 500
Tag density
b
chr2:62325146-62365146
10kbp
shBrd8
shLuc
D6
D4
D2
D0
Dpp4
Gm13561
chr9:78348177-78388177
Gsta2
Dppa5a
Ooep
chrX:86171764-86211764
Nr0b1
d
chr4:103599359-103639359
Dab1
chr2:85116225-85156225
Aplnr
chr5:147614418-147654418
Flt1
c
Dpp4
ESC 2iL
ESC S+L
EpiSC
0
500
Dppa5a
50000
Nr0b1
1000
Normalized tag count
e
Dab1
Aplnr
100
Flt1
2000

Figure S4

Figure S4. Chromatin accessibility during the primed-to-naïve transition.

- **a** Pileup heatmaps for ATAC-seq accessibility data showing the clusters of chromatin loci.
- **b** Genome views at naïve-specific genes' loci showing the ATAC-seq data in EpiSCs undergoing a primed-to-naïve transition in cells transfected with the indicated shRNA.
- **c** Bar charts showing gene expression or naïve-specific genes from RNA-seq data in ESCs grown in groundstate or naïve conditions (2iL or SL) and primed conditions (EpiSCs). Data is from **Figure S3a**.
- **d** Genome views at primed-specific genes' loci showing the ATAC-seq data in EpiSCs undergoing a primed-to-naïve transition in cells transfected with the indicated shRNA.
- **e** Bar charts showing gene expression or naïve-specific genes from RNA-seq data in ESCs grown in groundstate or naïve conditions (2iL or SL) and primed conditions (EpiSCs). Data is from **Figure S3a**.

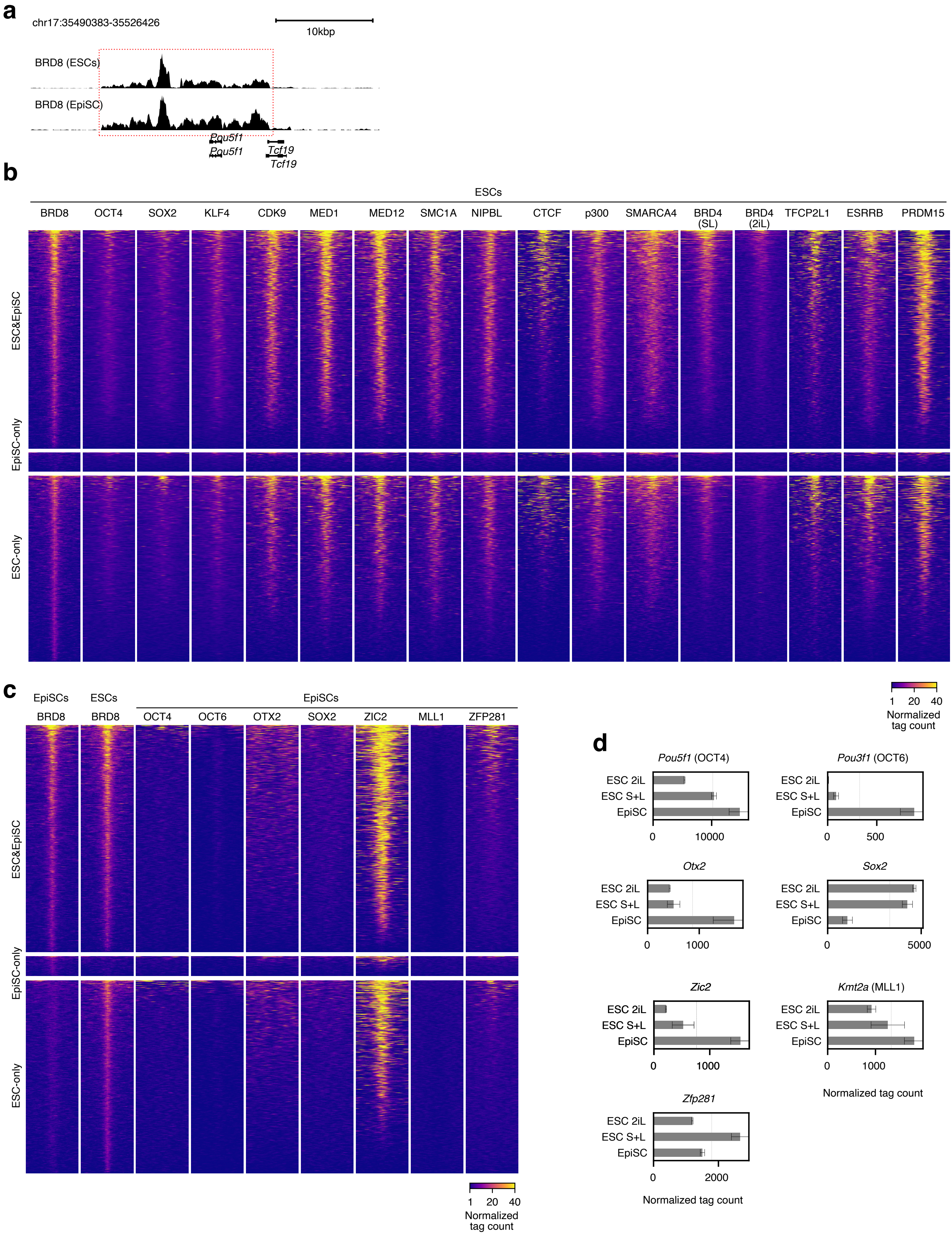

a
chr17:35490383-35526426
10kbp
BRD8 (ESCs)
BRD8 (EpiSC)
Pou5f1
Pou5f1
Tcf19
Tcf19
b
ESCs
BRD8
OCT4
SOX2
KLF4
CDK9
MED1
MED12
SMC1A
NIPBL
CTCF
p300
SMARCA4
BRD4 (SL)
BRD4 (2iL)
TFCP2L1
ESRRB
PRDM15
ESC&EpiSC
EpiSC-only
ESC-only
1 20 40
Normalized tag count
c
EpiSCs
ESCs
EpiSCs
BRD8
BRD8
OCT4
OCT6
OTX2
SOX2
ZIC2
MLL1
ZFP281
ESC&EpiSC
EpiSC-only
ESC-only
1 20 40
Normalized tag count
d
Pou5f1 (OCT4)
ESC 2iL
ESC S+L
EpiSC
0 10000
Pou3f1 (OCT6)
0 500
Otx2
0 1000
Sox2
0 5000
Zic2
0 1000
Kmt2a (MLL1)
0 1000
Normalized tag count
Zfp281
0 2000
Normalized tag count

Figure S5

Figure S5. BRD8 co-binds with cell type-specific transcription factors in ESCs and EpiSCs.

- **a** Genome view of the *Pou5f1* locus and the CUT&Tag data. As OG2 mice and cells contain multiple copies of the *Pou5f1* locus they result in an enhanced 'background' at the *Pou5f1* locus (marekd with the dotted red box) as the multiple copies contribute increased background. This confirms the cells are derived from OG2 mice.
- **b** Heatmap pileups of binding at BRD8 loci in ESCs for selected transcription and epigenetic factors in mESCS. Data is from OCT4 ESC, SOX2 ESC, KLF4 ESC, TFCP2L1 ESC, ESRRB ESC (all GSE11431) [9], and PRDM15 ESC (GSE73692) [10].
- **c** Heatmap pileups of binding at BRD8 loci in EpiSCs (and BRD8 in ESCs). Data is from: OTX2 EpiSC, ZIC2 EpiSC, SOX2 EpiSC, OCT4 EpiSC, OCT6 EpiSC (All GSE74636) [11], MLL1 EpiSC (GSE73992) [12], and ZFP281 EpiSC (GSE93042) [13].
- **d** Bar charts showing gene expression of the transcription or epigenetic factors in **panel B** from RNA-seq data in ESCs grown in groundstate or naïve conditions (2iL or SL) and primed (EpiSCs). Data is from **Figure S3a**.

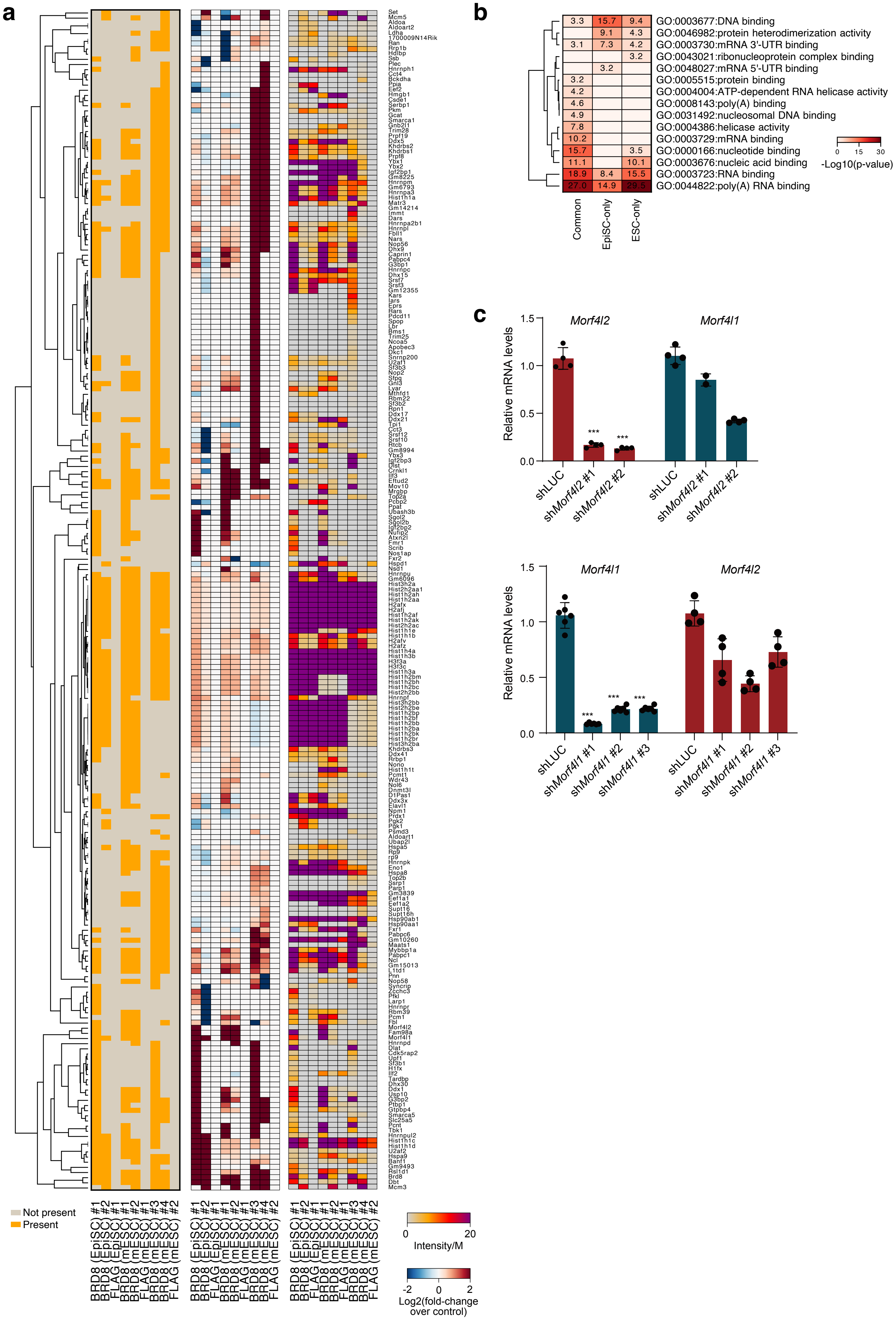
a
Not present
Present
BRD8 (EpiSC) #1
BRD8 (EpiSC) #2
FLAG (EpiSC) #1
BRD8 (mESC) #1
BRD8 (mESC) #2
FLAG (mESC) #1
BRD8 (mESC) #3
BRD8 (mESC) #4
FLAG (mESC) #2
Intensity/M
0
20
Log2(fold-change over control)
-2
0
2
b
3.3 15.7 9.4 GO:0003677:DNA binding
9.1 4.3 GO:0046982:protein heterodimerization activity
3.1 7.3 4.2 GO:0003730:mRNA 3'-UTR binding
3.2 GO:0043021:ribonucleoprotein complex binding
3.2 GO:0048027:mRNA 5'-UTR binding
3.2 GO:0005515:protein binding
4.2 GO:0004004:ATP-dependent RNA helicase activity
4.6 GO:0008143:poly(A) binding
4.9 GO:0031492:nucleosomal DNA binding
7.8 GO:0004386:helicase activity
10.2 GO:0003729:mRNA binding
15.7 3.5 GO:0000166:nucleotide binding
11.1 10.1 GO:0003676:nucleic acid binding
18.9 8.4 15.5 GO:0003723:RNA binding
27.0 14.9 29.5 GO:0044822:poly(A) RNA binding
Common
EpiSC-only
ESC-only
0 15 30
-Log10(p-value)
c
Morf4l2
Morf4l1
Relative mRNA levels
***
***
shLUC
shMorf4l2 #1
shMorf4l2 #2
shLUC
shMorf4l2 #1
shMorf4l2 #2
Morf4l1
Morf4l2
Relative mRNA levels
***
***
***
shLUC
shMorf4l1 #1
shMorf4l1 #2
shMorf4l1 #3
shLUC
shMorf4l1 #1
shMorf4l1 #2
shMorf4l1 #3

Figure S6

Figure S6. BRD8 interacts with epigenetic factors in EpiSCs

- **a** Heatmap of the interacting proteins from Co-IP/MS data in EpiSCs and ESCs using antibodies against BRD8, or an anti-FLAG antibody as a control. The left heatmap shows the 'present' 'not present' call for the indicated protein in the indicated condition. The middle heatmap shows the detected protein's fold-change. The rightmost heatmap shows the intensity of detection. The full table is in **Table S2**.
- **b** Gene ontology (molecular function) for all proteins detected by Co-IP/MS in the cell types as defined in **Figure 4f**).
- **c** RT-qPCR bar charts showing the knockdown efficiency of the *Morf4l1* and *Morf4l2* shRNAs used in this study. Cells were transfected with the indicated shRNA. The experiment was repeated three times. Error bars are standard error of the mean. * indicates significance <0.05, ** < 0.01 *** < 0.001. Significance is from two-sided unpaired Student's t-test.

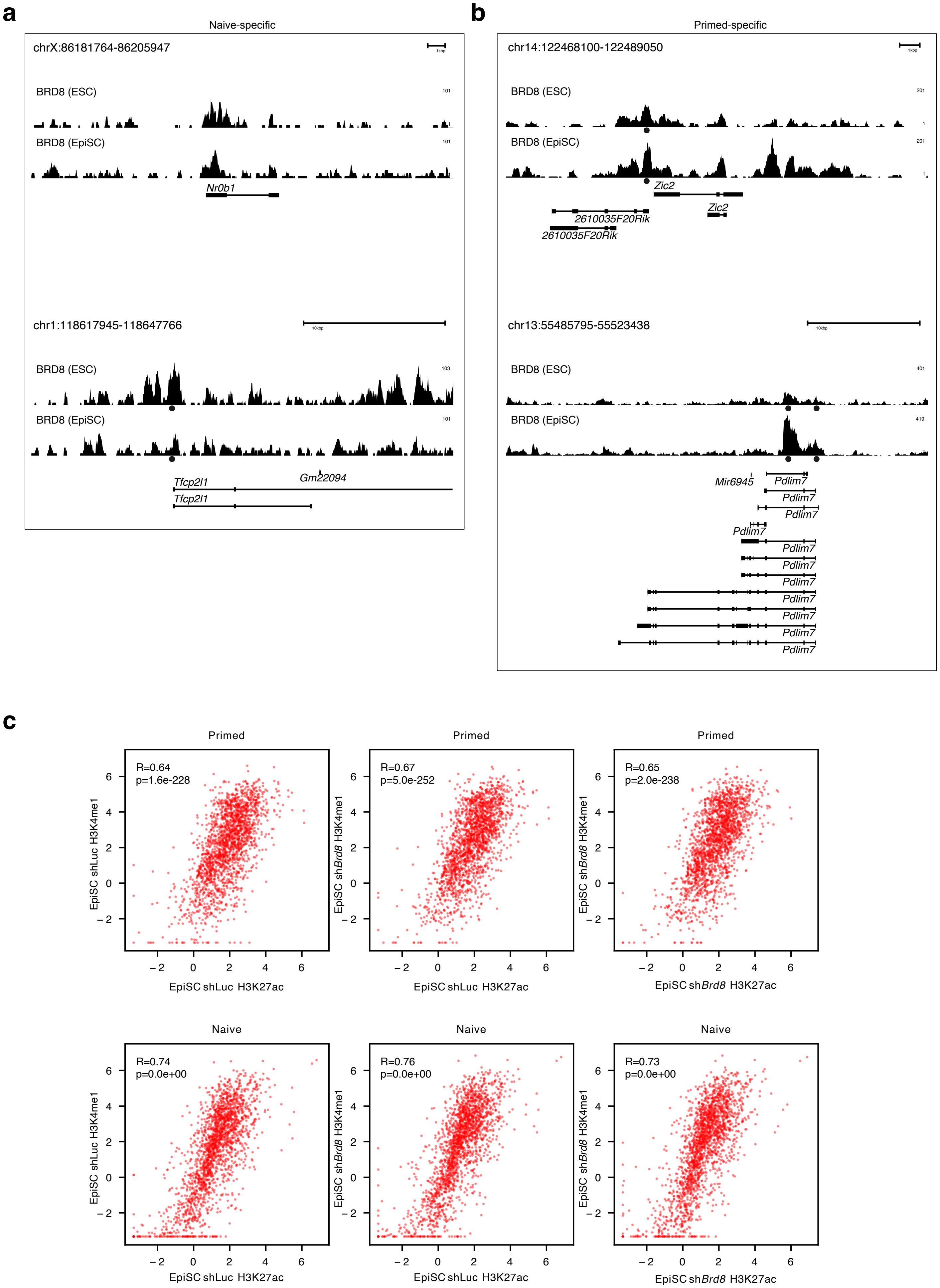
a
Naive-specific
chrX:86181764-86205947
1kbp
BRD8 (ESC)
BRD8 (EpiSC)
Nr0b1
chr1:118617945-118647766
10kbp
BRD8 (ESC)
BRD8 (EpiSC)
Tfcp2l1
Gm22094
Tfcp2l1
b
Primed-specific
chr14:122468100-122489050
1kbp
BRD8 (ESC)
BRD8 (EpiSC)
Zic2
2610035F20Rik
Zic2
2610035F20Rik
chr13:55485795-55523438
10kbp
BRD8 (ESC)
BRD8 (EpiSC)
Mir6945
Pdlim7
c
Primed
R=0.64
p=1.6e-228
EpiSC shLuc H3K4me1
EpiSC shLuc H3K27ac
Primed
R=0.67
p=5.0e-252
EpiSC shBrd8 H3K4me1
EpiSC shLuc H3K27ac
Primed
R=0.65
p=2.0e-238
EpiSC shBrd8 H3K4me1
EpiSC shBrd8 H3K27ac
Naive
R=0.74
p=0.0e+00
EpiSC shLuc H3K4me1
EpiSC shLuc H3K27ac
Naive
R=0.76
p=0.0e+00
EpiSC shBrd8 H3K4me1
EpiSC shLuc H3K27ac
Naive
R=0.73
p=0.0e+00
EpiSC shBrd8 H3K4me1
EpiSC shBrd8 H3K27ac

Figure S7

Figure S7. Example genome views at naïve and primed-specific genes.

- **a** Genome view of two naïve-specific genes, *Nr0b1* and *Tfcp2l1*, showing the BRD8 binding track (in black).
- **b** Genome view of two primed-specific genes, *Zic2* and *Pdlim7*, showing the BRD8 binding track (in black).
- **c** Scatter plots showing the levels of H3K4me1 versus H3K27me3 across the transcript bodies of primed and naïve-specific genes. X and Y axis are the log2 of the average read density from 300 bp 3' of the transcript start site (TSS) and 300 bp 5' of the transcription termination site in the indicated knockdowns in EpiSCs.

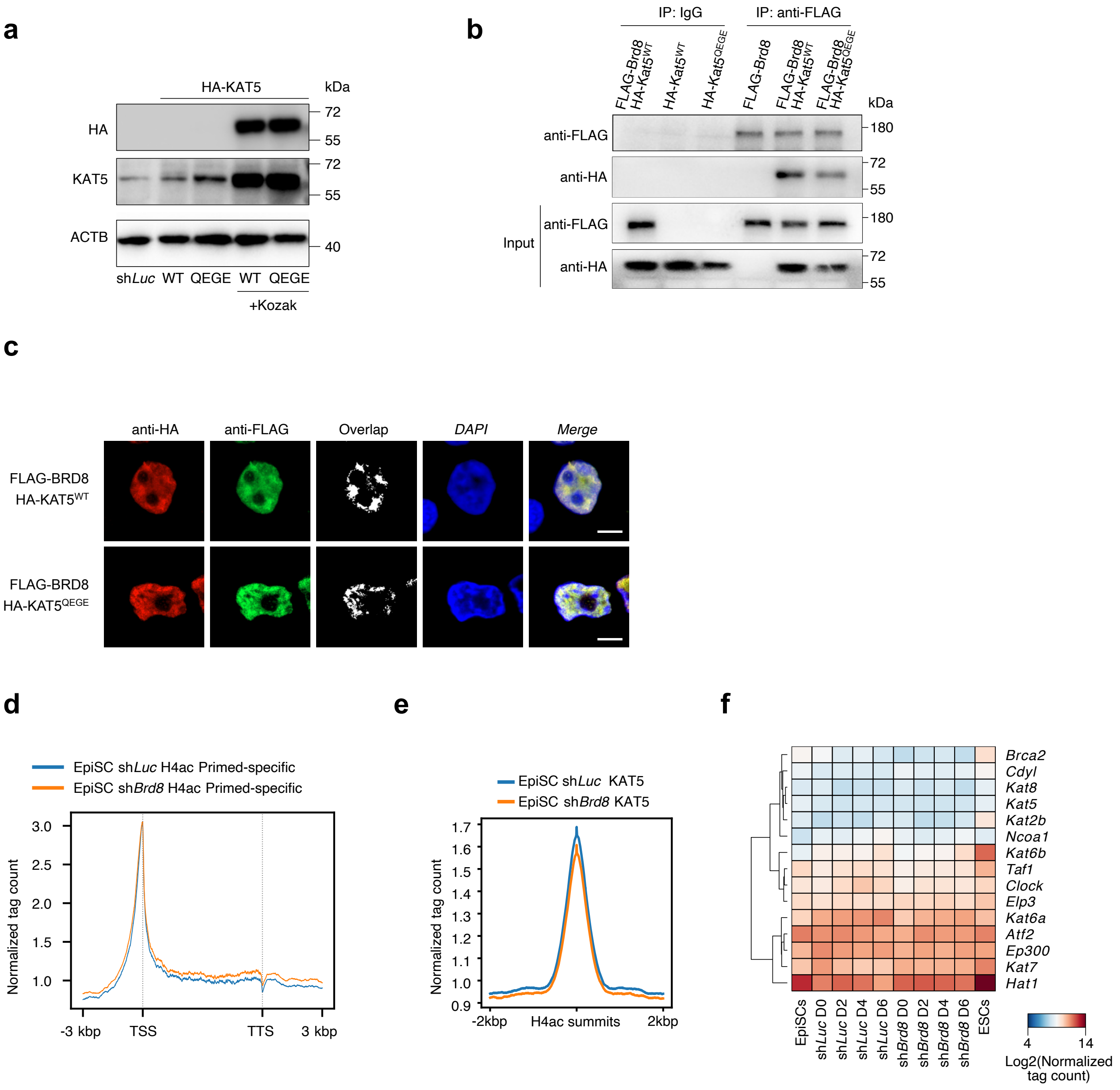
a
HA-KAT5
kDa
HA
KAT5
ACTB
72
55
40
shLuc WT QEGE WT QEGE
+Kozak
b
IP: IgG
IP: anti-FLAG
FLAG-Brd8 HA-Kat5WT
HA-Kat5WT
HA-Kat5QEGE
FLAG-Brd8
FLAG-Brd8 HA-Kat5WT
FLAG-Brd8 HA-Kat5QEGE
anti-FLAG
anti-HA
Input
180
c
anti-HA
anti-FLAG
Overlap
DAPI
Merge
FLAG-BRD8 HA-KAT5WT
FLAG-BRD8 HA-KAT5QEGE
d
EpiSC shLuc H4ac Primed-specific
EpiSC shBrd8 H4ac Primed-specific
Normalized tag count
3.0
2.5
2.0
1.5
1.0
-3 kbp
TSS
TTS
3 kbp
e
EpiSC shLuc KAT5
EpiSC shBrd8 KAT5
1.7
1.6
1.4
1.3
1.2
1.1
0.9
-2kbp
H4ac summits
2kbp
f
Brca2
Cdyl
Kat8
Kat5
Kat2b
Ncoa1
Kat6b
Taf1
Clock
Elp3
Kat6a
Atf2
Ep300
Kat7
Hat1
EpiSCs
shLuc D0
shLuc D2
shLuc D4
shLuc D6
shBrd8 D0
shBrd8 D2
shBrd8 D4
shBrd8 D6
ESCs
4
14
Log2(Normalized tag count)

Figure S8

Figure S8. BRD8 interacts with catalytic null KAT5.

- **a** Western blot for the overexpression of KAT5 wildtype (WT) or a catalytic-null $KAT5^{QEGE}$ ($KAT5^{Q377E/G380E}$) [14].
- **b** Western blot of a co-immunoprecipitation of FLAG-tagged BRD8 or HA-tagged KAT5 (either WT or QEGE catalytic mutant forms) in 293T cells overexpressing the indicated vectors.
- **c** Immunofluorescence staining in 293T cells transfected with the indicated vectors. Scale bar = 5 μm.
- **d** Pileup of H4ac CUT&Tag data across primed-specific genes in EpiSCs transfected with an shRNA targeting *Luc* or *Brd8*.
- **e** Pileup of KAT5 at all H4ac summits in EpiSCs transfected with an shRNA targeting *Luc* or *Brd8*.
- **f** Heatmap showing the expression of known H4 acetyltransferase catalytic enzymes in EpiSCs.

Supplementary Figure 1c

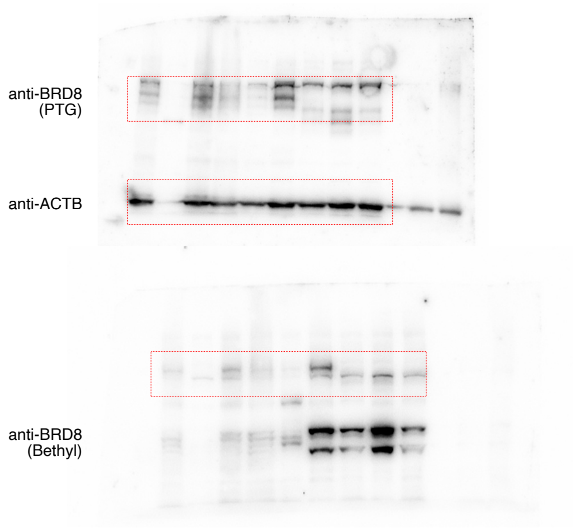

Supplementary Figure 8a

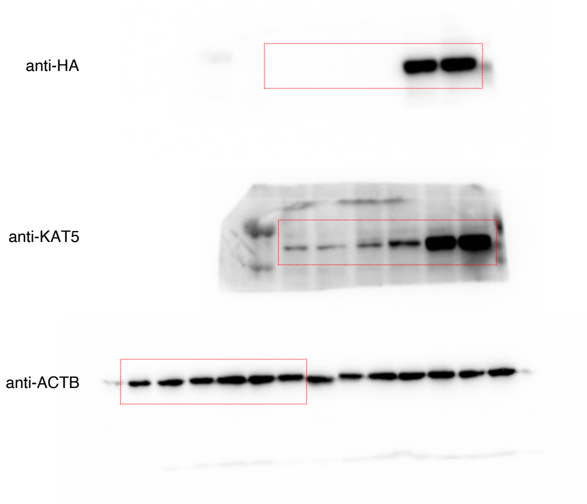

Figure 7a, b

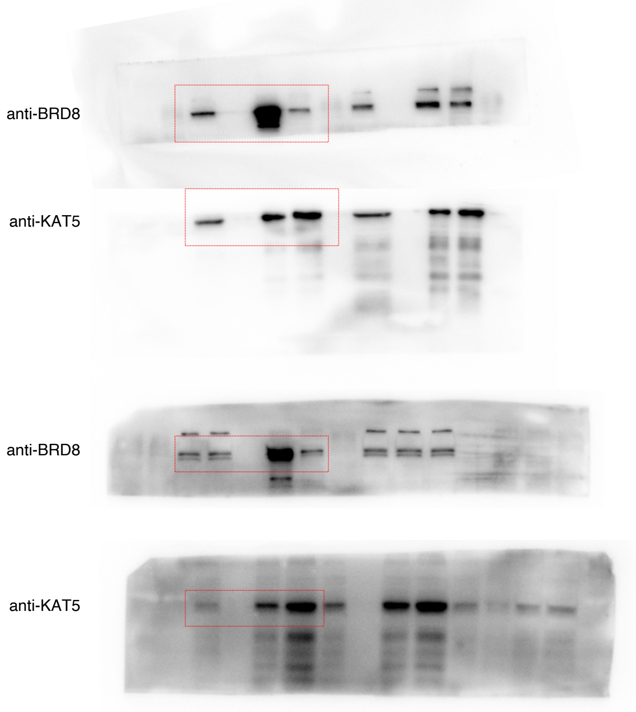

Figure 4h

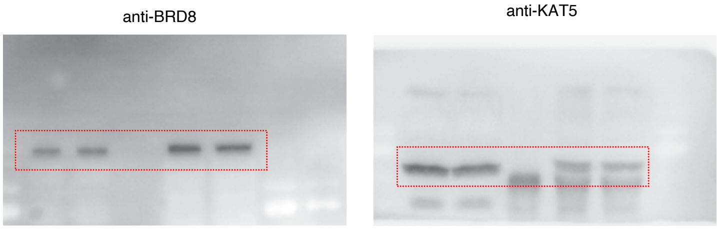

Figure 5a

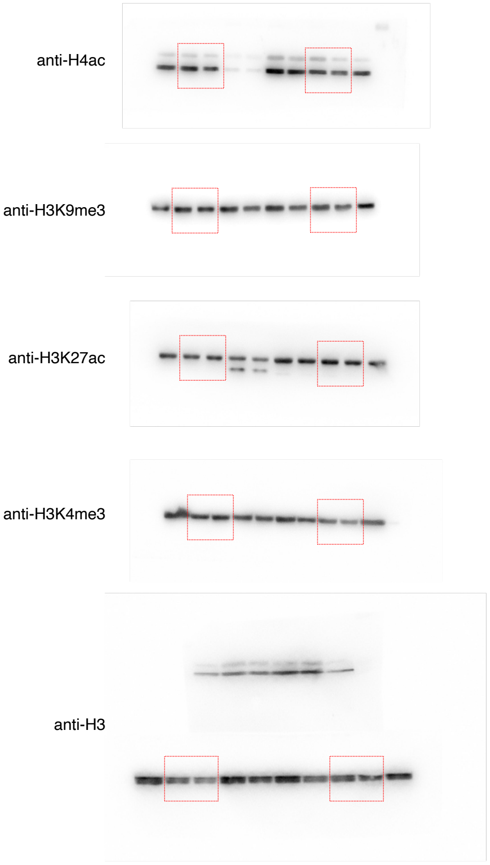

Supplementary Figure 8b

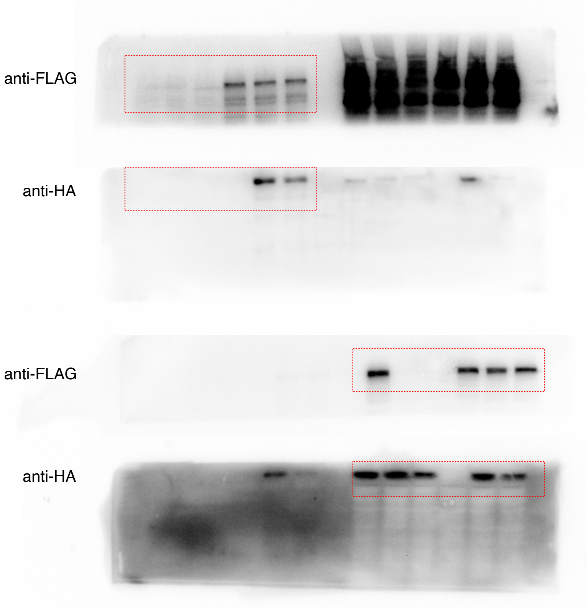

Supplement: Supplementary file 1 — Supporting Information [file ADVS-12-2409160-s003.pdf]
